# Investigating the Cell Entry Mechanism, Disassembly, and Toxicity of the Nanocage PCC-1: Insights into Its Potential as a Drug Delivery Vehicle

**DOI:** 10.1021/jacs.3c09918

**Published:** 2023-12-09

**Authors:** Zhifeng Xiao, Hengyu Lin, Hannah F. Drake, Joshua Diaz, Hong-Cai Zhou, Jean-Philippe Pellois

**Affiliations:** † Department of Biochemistry and Biophysics, Texas A&M University, College Station, Texas 77843, United States; ‡ Department of Chemistry, Texas A&M University, College Station, Texas 77843, United States

## Abstract

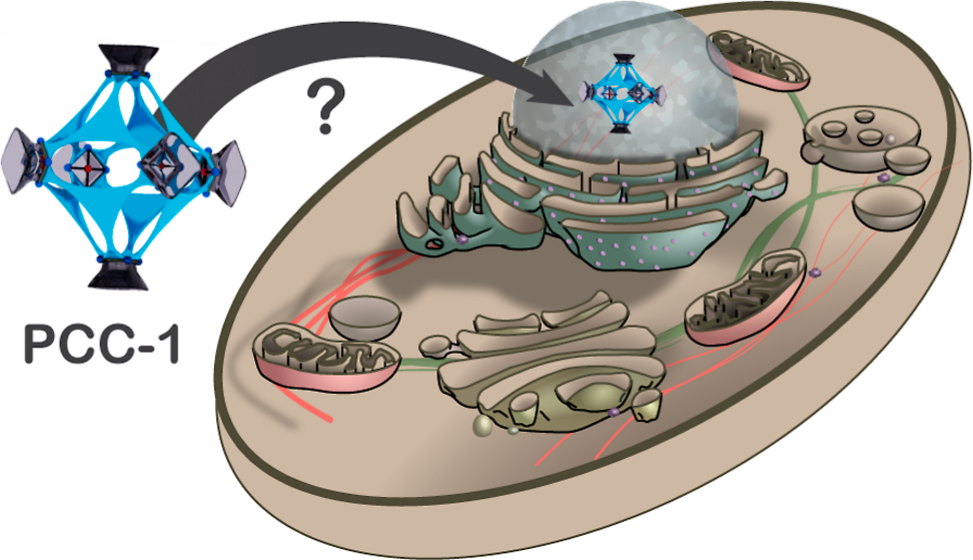

The porous coordination cage PCC-1 represents a new platform potentially useful for the cellular delivery of drugs with poor cell permeability and solubility. PCC-1 is a metal–organic polyhedron constructed from zinc metal ions and organic ligands through coordination bonds. PCC-1 possesses an internal cavity that is suitable for drug encapsulation. To better understand the biocompatibility of PCC-1 with human cells, the cell entry mechanism, disassembly, and toxicity of the nanocage were investigated. PCC-1 localizes in the nuclei and cytoplasm within minutes upon incubation with cells, independent of endocytosis and cargo, suggesting direct plasma membrane translocation of the nanocage carrying its guest in its internal cavity. Furthermore, the rates of cell entry correlate to extracellular concentrations, indicating that PCC-1 is likely diffusing passively through the membrane despite its relatively large size. Once inside cells, PCC-1 disintegrates into zinc metal ions and ligands over a period of several hours, each component being cleared from cells within 1 day. PCC-1 is relatively safe for cells at low micromolar concentrations but becomes inhibitory to cell proliferation and toxic above a concentration or incubation time threshold. However, cells surviving these conditions can return to homeostasis 3–5 days after exposure. Overall, these findings demonstrate that PCC-1 enters live cells by crossing biological membranes spontaneously. This should prove useful to deliver drugs that lack this capacity on their own, provided that the dosage and exposure time are controlled to avoid toxicity.

## Introduction

Small-molecule drugs (<1 kDa) are the most common therapeutic modalities for treating numerous human diseases.^1–5^ The investment in small-molecule drug discovery was approximately $32 billion in 2020, and it is expected to reach $51 billion by 2026.^6^ Compared to biologics (proteins and nucleic acids), small-molecule drugs are advantageous because of their relatively low cost, large production scale, and chemical stability. Small-molecule drugs can also be administered by convenient oral delivery, cross biological barriers, diffuse in the bloodstream, and quickly achieve peak concentrations at the site of action. For small-molecule drugs that target intracellular processes, a critical step in their therapeutic effects involves the penetration of cells. At a molecular level, this typically involves the passive diffusion of the drug across lipid bilayers.^7^ In turn, this requires that the lipophilicity of the small-molecule drug falls within a relatively narrow window.^8^ Specifically, a molecule that is too hydrophilic will not cross the hydrophobic region of a lipid bilayer and, as a result, will not penetrate the cell membrane. In contrast, a molecule that is too hydrophobic may insert into a lipid bilayer but remain trapped in a membrane. In addition, high-hydrophobicity molecules have poor solubility, reducing efficacy and limiting use.^9,10^ Overall, designing drugs that have adequate cell permeability and solubility remains a challenge, and these constraints represent significant bottlenecks in the drug discovery process. To address cell permeability and solubility issues, medicinal chemists typically explore the chemical space around lead compounds, often screening large libraries of chemical modifications around a parent structure.^2,11–13^ An alternative is to utilize a drug delivery system that can encapsulate drugs and circumvent poor solubility.^10,14,15^ An example consists of liposomal formulations.^16–19^ Notably, encapsulation systems are relatively large and are internalized in cells by endocytic uptake.^15,20^ Endocytosis may, in turn, limit biodistribution. For instance, once endocytosed, drug payloads are often trapped inside endosomes and fail to reach their intracellular targets.^21–23^

Porous coordination cages (PCCs) have recently been reported as new potential drug delivery systems.^24–31^ PCCs are metal–organic polyhedra constructed from metal ions and organic ligands through coordination bonds.^32–34^ PCCs exist in solution as discrete cage-like molecules^33,35,36^ and possess an internal cavity allowing host–guest interactions and drug molecules encapsulation.^37^ The internal cavities of PCCs are walled by organic linkers and are often nonpolar, thereby suitable for binding small hydrophobic drug molecules.^38,39^ The cage, PCC-1, based on a tetranuclear sulfonylcalix[4]arene-Zn cluster, enters the nucleus of cells, delivers a hydrophobic topoisomerase I inhibitor [camptothecin (CPT)] in this organelle, and improves the anticancer activity of this drug in vitro and in vivo.^26^ Consistent with these results, PCC-1 encapsulation also changed the localization of a fluorescent probe, Nile Red, from the cytoplasm to the nucleus.^26^ Other PCCs, ZnPMTC^31^ and Zn-NH-pyr,^30^ localize in the lysosome and mitochondria, respectively. In turn, these cages deliver hydrophobic anti-inflammatory agents into macrophages and reduce joint inflammation in rat models. These results highlight that PCCs are potentially valuable for organelle-directed delivery of drugs with suboptimal lipophilicity. Several fundamental aspects of PCCs need to be elucidated to expand on these exciting results. For instance, PCCs rely on metal chelation with organic ligands to form their structures. The impact these components, metal ions, and organic ligands have on cells is unclear. The introduction of metal in cells can perturb homeostasis and lead to toxicity.^40^ Notably, zinc coordinated in the cage structure will not impact cells to the same extent as the zinc released upon cage degradation.^41^ The same idea extends to other cage components, namely, panel and vertex ligands. Hence, cellular responses and toxicity are likely intimately related to the decomposition processes. Likewise, the mechanisms by which PCCs enter cells and how long PCCs stay inside cells contribute to modulating cellular responses. In this report, we therefore seek to better understand the biocompatibility of PCC with human cells. We used PCC1 and tissue cultures as model systems. We ask the following questions: (1) can PCC-1 bring cell impermeable cargos into cells, (2) how does PCC-1 enter cells and what are the kinetics of this process, (3) how quickly does PCC-1 decompose in cells, and (4) how do cells respond to PCC-1 entry and decomposition?

## Results

### Cargo Encapsulation

**Figure 1:**
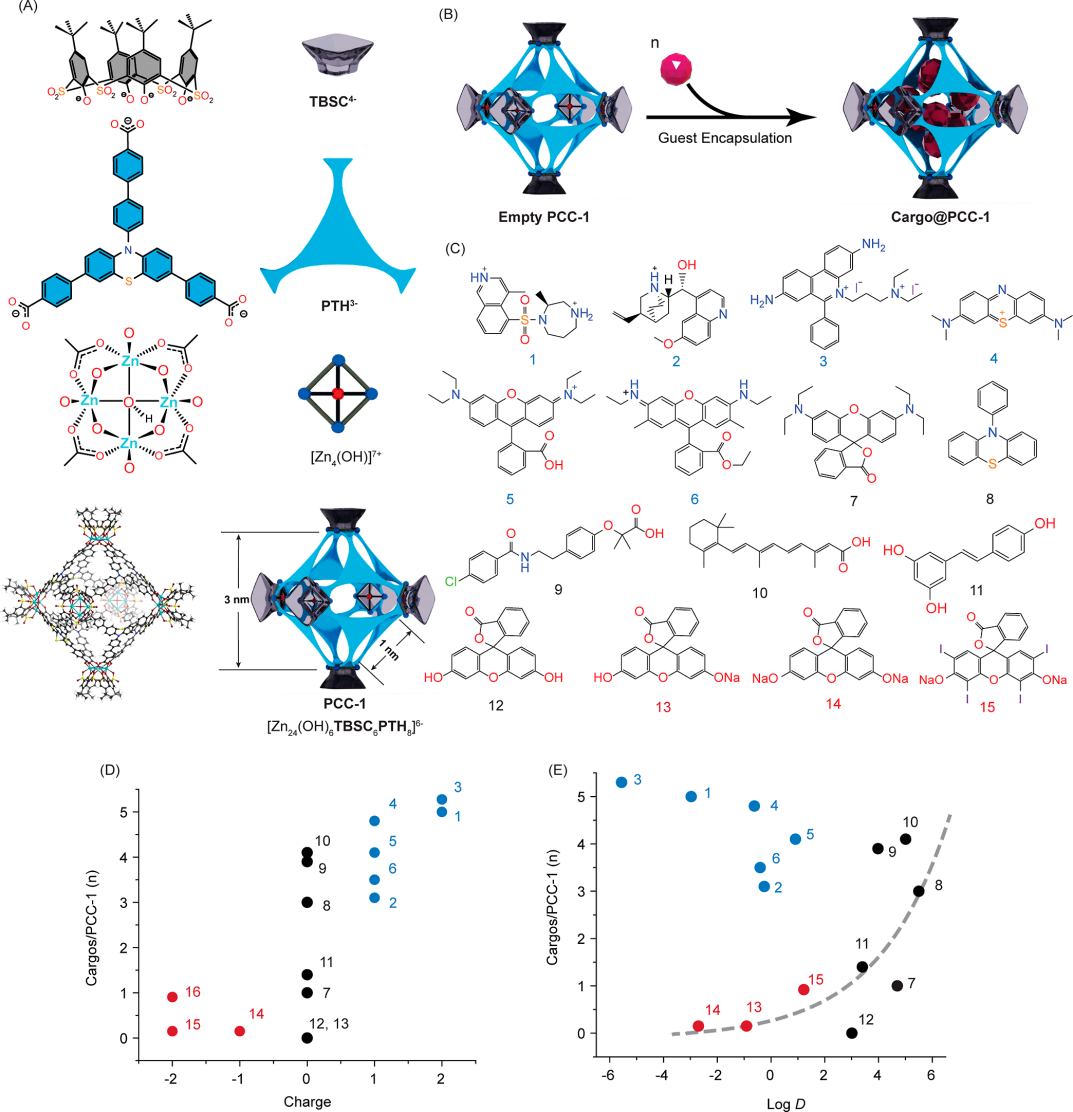
Structure of PCC-1 and guest encapsulation. (A) Building blocks and structure of PCC-1. PCC-1 is assembled from a calixarene-based ligand (TBSC^4–^), a fluorescent ligand (PTH^3–^), and a tetranuclear Zn-based cluster [Zn (OH)^7+^]. The crystal structure of PCC-1 is represented, along with an illustration highlighting the dimensions of the nanocage. (B) Schematic representation of guest encapsulation with PCC-1. (C) Structures of guest molecules tested for PCC-1 encapsulation. (D) Correlation between charges of guest molecules and the number of cargo molecules per PCC-1 cage. (E) Correlation between the computed Log *D* of guest molecules and the number of cargo molecules per PCC-1 cage. The fitting curve (gray dotted line) between Log *D* and cargo molecules per PCC-1 among neutral and anionic cargoes is fit using a two-parameter exponential mathematical model (*y* = *ae*^*bx*^).

PCC-1 is an octahedral cage with an internal cavity of 3 nm width and openings of 1 nm (Figure 1A).^26^ The assembly of PCC-1 is through the formation of coordination bonds between Zn^2+^ ions and two kinds of ligands, carboxylate-based panel ligands (H_3_PTH) and phenolate-based vertex ligands (H_4_TBSC) (synthesis in Figure S1). PCC-1 carries an overall charge of −6 and is expected to form electrostatic interactions with cationic guest molecules. The internal cavity of PCC-1 is surrounded by eight hydrophobic panel ligands, H_3_PTH, and is capped by six vertices containing central μ^4^-OH groups. With the μ^4^-OH group pointing toward the center of the cavity, hydrogen bonding can form between the μ^4^-OH group and the encapsulated guest molecules. The structural features of the internal cavity make it possible to encapsulate molecules with various properties (Figure 1B). To identify probes useful for investigating cell entry, several fluorophores were tested as potential payloads for PCC-1 (Figure 1C). Several nonfluorescent drugs were also included as controls. The guest molecule encapsulation was carried out by submerging the activated PCC-1 crystals into an acetonitrile solution of the cargo molecules (adsorption kinetics in Figure S2). The encapsulation ratios of PCC-1 to cargo molecules were extrapolated from the reduction of cargo molecules in solution and the mass of PCC-1 crystals. As shown in Figure 1D, the tested cationic cargo molecules exhibit overall higher loading than the neutral cargo molecules, presumably due to the anionic nature of PCC-1. Among positively charged cargo molecules, lipophilicity, as measured by the pH-dependent octanol–water partition coefficient (Log *D*), does not affect the loading. In contrast, the loading of neutral cargo molecules generally increases with lipophilicity (Figure 1E). Anionic fluorophores were not encapsulated. Overall, these results indicate that PCC-1 favors the binding of cationic cargo molecules and hydrophobic neutral molecules. For subsequent assays, we used methylene blue (MB, molecule 4 in Figure 1C) as a fluorescent model cargo.

To evaluate the stability of the nanocage before cell experiments, both PCC-1 and MB@PCC-1 were incubated in PBS at pH levels of 6, 7, and 8 over 7 days. We measured the release of H3PTH and MB using high-performance liquid chromatography. The data show that PCC-1’s stability is influenced by pH levels (Figure S3). While PCC-1 remains stable at acidic pH, it starts to break down at pH 7 and 8. The decomposition rate of empty PCC-1 is modest, with around 5% decomposition observed after 24 h at pH 7 and 32% at pH 8. Intriguingly, the MB seems to bolster the stability of its PCC-1 host. Specifically, MB@PCC-1 shows less than 1.5% decomposition at pH 7 and under 3% decomposition at pH 8 over a 24 h period. This may imply that MB either prevents water from accessing the PCC-1 cavity or forms stabilizing weak bonds with PCC-1 components. Collectively, our findings confirm that PCC-1 maintains stability in aqueous buffers over extended durations. Based on this evidence, we rationalized that degradation of these compounds outside of cells would be unlikely to confound results if relatively short incubation times were used. In subsequent experiments with cells, we therefore focused on testing 1–5 h incubations.

### PCC-1 Translocation into Live Cells and Cargo Transport

**Figure 2:**
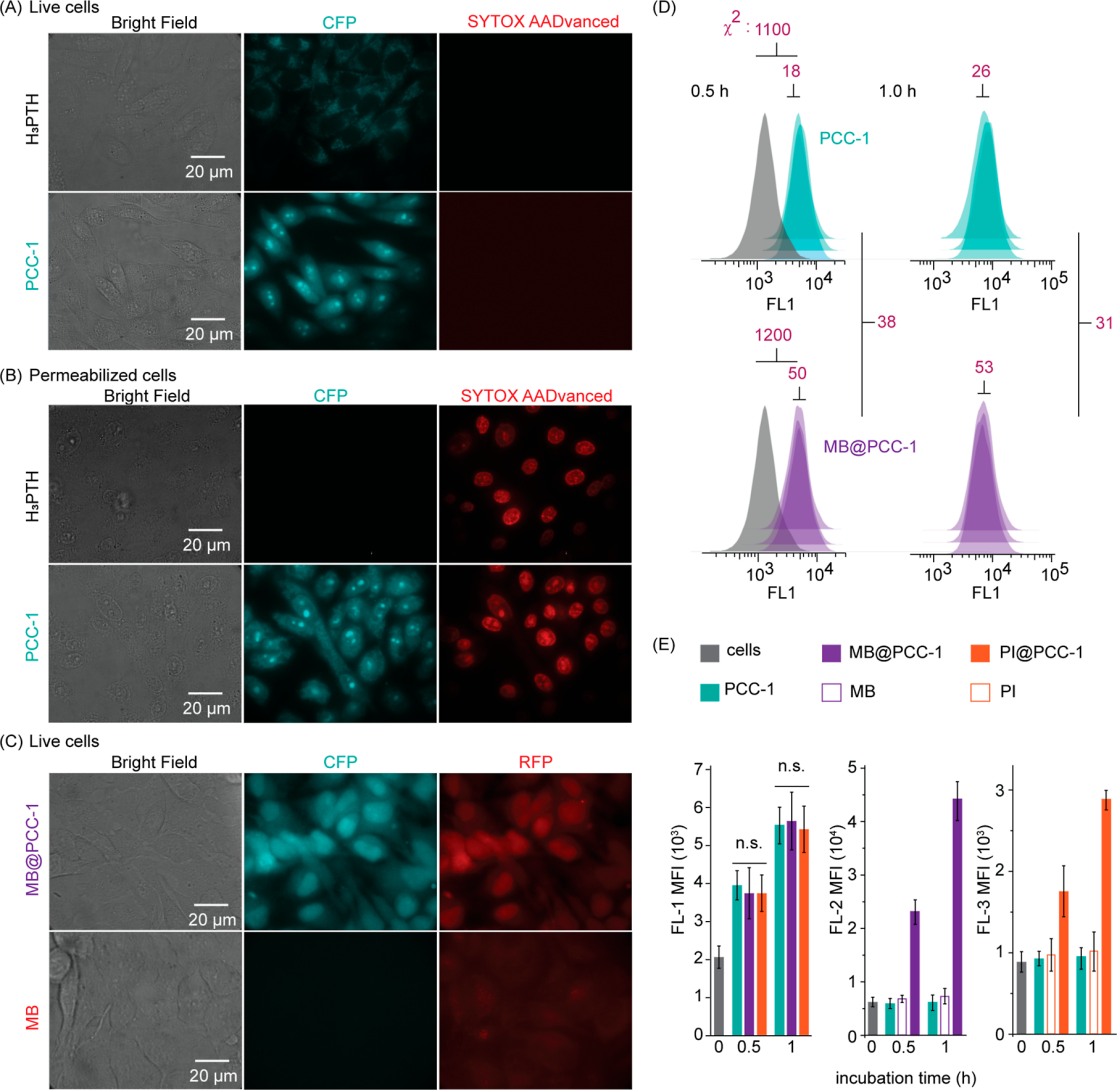
Intracellular localization and membrane translocation of PCC-1 in CHO-K1 cells. (A) Fluorescence microscopic images of the intracellular localizations of PCC-1 (1.2 μM) and its ligand H_3_PTH (10 μM) in live cells. The CFP channel detects the fluorescence of H_3_PTH, as a free ligand or integrated in PCC-1. SYTOX AADvanced is a cell-impermeant nucleic acid dye that stains cells with compromised plasma membranes but that does not stain live cells. (B) Fluorescence microscopic images of the intracellular localization of PCC-1 and its ligands H_3_PTH in permeabilized cells. SYTOX AADvanced staining establishes that cells are permeabilized. (C) Fluorescence microscopic images of the intracellular localization of PCC-1 with MB. The RFP channel shows the localizations of MB. (D) Flow cytometry profiles of cells treated with PCC-1 (1.2 μM, green) and MB@PCC-1 (1.2 μM, purple) for 0.5 and 1.0 h. Gray populations correspond to untreated cells. Each panel shows biological triplicates. The χ^2^-test scores for the comparison of populations are provided. (E) Comparison of the mean fluorescence intensity (MFI) of cells treated with PCC-1, MB@PCC-1, and PI@PCC-1 (1.2 μM) as a function of time (0.5 or 1 h incubation) in the Fl-1 channel (corresponding to the fluorescence of PCC). The MFI in the FL-2 channel (corresponding to the fluorescence of MB) and FL-3 channel [corresponding to the fluorescence of propidium iodide (PI)] are also presented. Controls include incubations with PCC-1 alone (1.2 μM), MB alone (10 μM), and PI alone (10 μM). The data represent the average and corresponding standard deviations of the biological triplicates, with 4 × 10^4^ cells analyzed per replicate. n.s. = nonsignificant, *p* > 0.05.

The entry of PCC-1 into cells was characterized in the CHO-K1 cell line (epithelial). PCC-1 cell entry was monitored by exploiting the intrinsic fluorescence of the H_3_PTH ligand. Incubation of cells with H_3_PTH alone yielded weak intracellular fluorescence (Figure 2A, flow cytometry quantification in Figure S4). This fluorescence is localized in puncta, suggesting endocytosis and endosomal accumulation. In comparison, cells incubated with PCC-1 show diffuse blue fluorescence with distinct staining of nuclei and nucleoli, indicative of intracellular accumulation (as opposed to out-of-focus extracellular fluorescence). The fluorescence of cells was also approximately 15-fold higher when treated with PCC-1 (1.2 μM) than when treated with (PCC-1 containing 8 H3PTH ligands, a 10 μM concentration of free H3PTH was used) (Figure S4). PCC-1 staining is detected in the absence of SYTOX AADvanced Dead Cell staining (SYTOX). In turn, this indicates that the plasma membrane of these cells is not permeable and that the cells are not dead. Cells permeabilized by treatment with digitonin, a mild cell permeation reagent, were also used to test where H_3_PTH would localize if given access to the cell’s interior. Unlike live cells, permeabilized cells were readily stained by SYTOX. PCC-1 stained nuclei and nucleoli, as observed in live cells (albeit with higher contrast between nucleoli and nucleus, presumably because excess PCC-1 is washed away in permeabilized cells but not live cells) (Figure 2B). In contrast, H_3_PTH showed weak cytoplasmic staining, with no observable signal in nuclei or nucleoli. These results indicate that the nuclear and nucleolar fluorescence observed upon PCC-1 incubation is that of the nanocage and not the free H_3_PTH ligand. These data also indicate that the nanocage has an intrinsic affinity for nucleoli and nuclei over other cellular compartments.

To test whether this fluorescence signal corresponds to a form of PCC-1 that can encapsulate a payload, PCC-1 was loaded with MB (MB@PCC-1) as a red fluorescent cargo. Incubation of cells with MB alone led to weak fluorescent puncta, consistent with the accumulation of MB in endosomes. Notably, the fluorescence of MB does not colocalize with nuclei (Figure 2C). In contrast, cells incubated with MB@PCC-1 show nuclear localization of both the blue fluorescence signal of PCC-1 and the red fluorescence of MB, indicating that PCC-1 and MB enter the cells together. To establish whether PCC-1 and MB@PCC-1 differ in their intracellular penetration, the cellular fluorescence of PCC-1 was quantified by flow cytometry (PCC-1 was detected in the FL-1 channel). Cells were incubated for 0.5 or 1 h with PCC-1 and MB@PCC-1 at 1.2 μM. The χ^2^-test (Figure 2D) was utilized to compare the differences in the amount of PCC-1 taken up by cells between conditions.^42–44^ The χ^2^-test estimates the probability that a test population is statistically different from the control population. The χ^2^ scores between untreated cells and cells treated with PCC-1 or MB@PCC-1 were 1100 and 1200, respectively (Figure 2D). The χ^2^ scores between PCC-1 and MB@PCC-1 were 38 and 31 for 0.5 and 1 h treatment groups, respectively, i.e., less than the score for biological triplicates. Therefore, no statistical difference was observed between the amounts of fluorescence in cells for both PCC-1 and MB@PCC-1 at the two time points (Figure 2E). Notably, the uptake of MB by cells, detected in the FL-2 channel, was negligible when MB was incubated alone (10 μM). In contrast, the FL-1 and FL-2 signals increased with time when cells were incubated with MB@PCC-1 (1.2 μM), suggesting concurrent uptake of PCC-1 and MB (Figure 2E; PCC-1 alone does not contribute to FL-2).

To expand these results to other potential payloads, similar experiments were repeated with PI (compound 3 in Figure 1) and with PI@PCC-1 (Figure S5). PI is cell impermeable, and this fluorescent dye is commonly used to detect dead cells with damaged membranes. In our assays, incubation of PI with live cells yielded no staining, as detected by flow cytometry (Figures 2E and S5). In contrast, PI@PCC-1 leads to an increased uptake of PI into cells (we confirmed that this is achieved without killing cells using SYTOX green staining). The amounts of PI@PCC-1 taken up at the 0.5 and 1 h time points also match that of PCC-1 alone or MB@PCC-1 (Figure 2E). Overall, considering that MB or PI cannot enter cells efficiently without a facilitator (as shown by our controls), these combined data indicate that PCC-1 reaches the intracellular milieu and brings along cell-impermeable cargos like MB and PI. The consistency in cellular uptake between PCC-1, MB@PCC-1, and PI@PCC-1 indicates that the presence of the cargo does not alter the uptake mechanism or efficiency of the nanocage. This serves as an indirect but strong evidence that the cargo is housed within the cavity of PCC-1 during the uptake process (in contrast, the presentation of MB or PI on the surface of PCC-1 would likely alter the interactions with cells along with the rate and extent of transport). Bolstering this hypothesis, NMR spectroscopy analysis revealed exchange broadening of the MB resonances in MB@PCC-1 (Figure S11). Such broadening is a hallmark of constrained molecular dynamics, further substantiating the notion that MB is confined within the PCC-1 cage.^45^

### Mechanisms of PCC-1 Translocation

**Figure 3:**
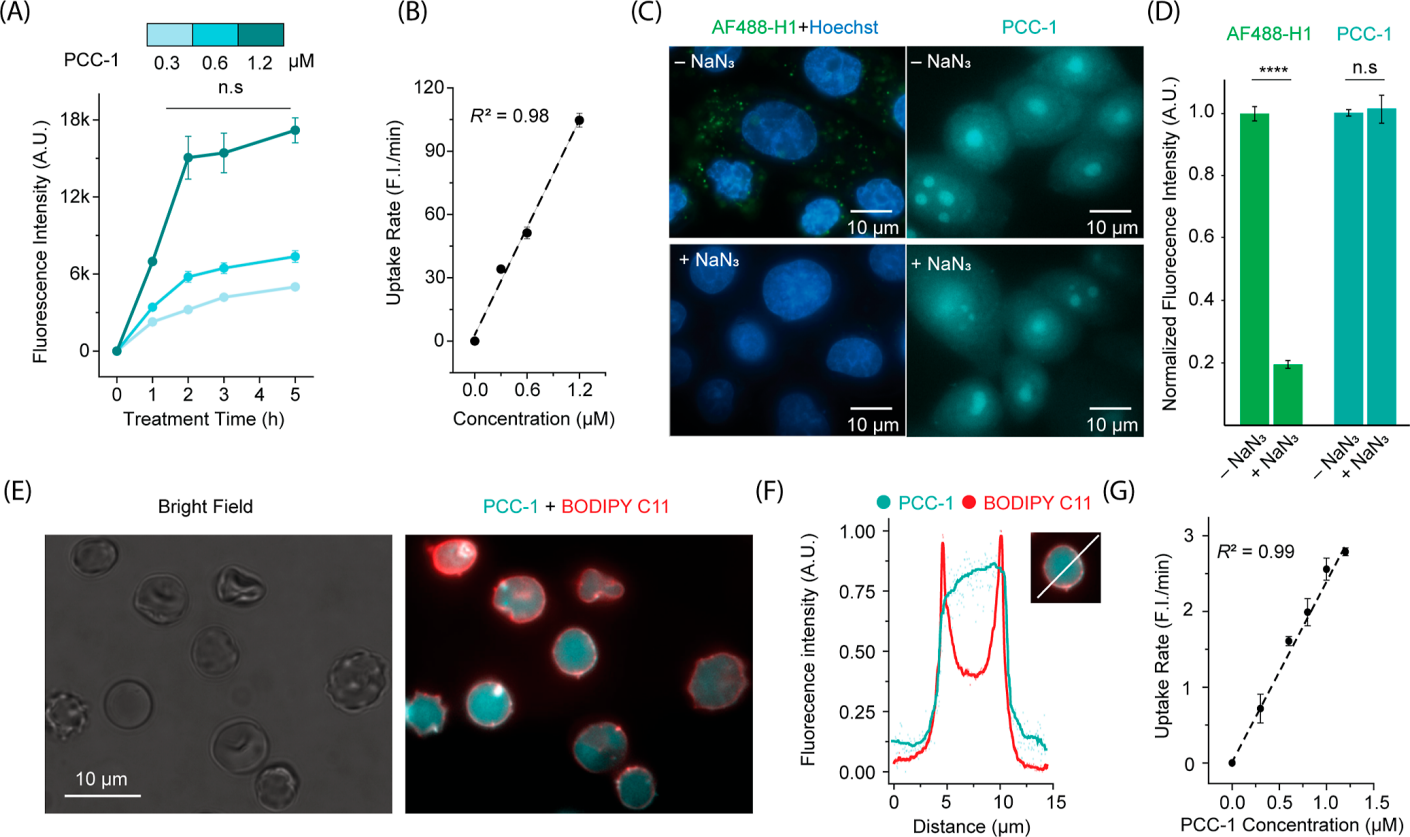
Intracellular transport of PCC-1. (A) Cellular uptake kinetics of PCC-1 into CHO-K1 under different treatment concentrations. The fluorescence intensities reported correspond to the MFI obtained of cell populations analyzed by flow cytometry. The data represent the average MFI and corresponding standard deviations of biological triplicates. Each replicate uses 4 × 10^4^ cells for analysis. (B) Cell uptake rate of PCC-1 in CHO-K1 cells as a function of PCC-1 concentration administered extracellularly. The MFI of cell populations were determined by flow cytometry. The data represent the average MFI and corresponding standard deviations obtained from biological triplicates, 4 × 10^4^ cells being analyzed per replicate. The black dotted line represents a linear fit (*R*^2^ = 0.98). (C) Fluorescence microscopic images of CHO-K1 cells treated with AF488-H1 and PCC-1, in the absence or presence of sodium azide (NaN_3_). Cell nuclei were stained with Hoechst for the AF488-H1 conditions. (D) Flow cytometry quantification of cell uptake of AF488-H1 and PCC-1 under different sodium azide treatment conditions. The MFI of sodium azide-treated groups were normalized to that of the sodium azide-free (–NaN_3_) groups. The significance level was evaluated by two-tailed *t* tests (****p < 0.0001; n.s., *p* > 0.05). (E) Fluorescence microscopic images of erythrocytes treated with PCC-1 (pseudocolored cyan) and BODIPY C11 (pseudocolored red) showing the cytosolic distribution of PCC-1 versus plasma membrane distribution of BODIPY C11. A bright-field image highlighting the morphology of cells is also provided. (F) Fluorescence intensity profiles and CFP and RFP channels, across a selected erythrocyte cell in (E) as shown in the inset. (G) Cell uptake rate in erythrocytes as a function of PCC-1 concentration administered extracellularly. The data represent the average intensities and corresponding standard deviations of 4 × 10^4^ cells. The black dotted line is a linear fit (*R*^2^ = 0.99).

The effects of incubation time and concentration on PCC-1 cell penetration were quantified by flow cytometry (Figure 3A,B). The intracellular fluorescence of PCC-1 increases within the first 2 h of incubation and reaches a plateau past this time point (Figure 3A). Notably, intracellular fluorescence is linearly proportional to the concentration of PCC-1 extracellularly administered in the concentration range tested (Figure 3B), indicating that transport is not saturable under the conditions used (higher concentrations were not used because of toxicity; see the next section). We next tested the effects of abolishing endocytosis on cell penetration. Cells were preincubated with sodium azide (40 mM, 5 h preincubation) to disrupt cellular processes requiring ATP (Figure 3C).^46–48^ The fluorescently labeled histone, AF488-H1, was used as a positive control. Sodium azide inhibited the endocytic uptake of AF488-H1, as evidenced by the absence of fluorescence puncta (Figure 3C) and a decrease in intracellular fluorescence, as detected by flow cytometry (Figure 3D). In contrast, sodium azide did not reduce the intracellular fluorescence accumulation of PCC-1. These results indicate that endocytic uptake is unnecessary for the cell penetration of PCC-1. Human red blood cells (RBCs), which lack the capacity for endocytosis,^49,50^ were used as an additional model to confirm these results. PCC-1 was incubated with RBCs at different concentrations for 1 h. RBCs were washed and then imaged by fluorescence microscopy. The red fluorescent dye BODIPY C11 was used to stain the plasma membranes of RBCs (Figure 3E). While the BODIPY C11 signal was restricted to the surface of RBCs, the blue fluorescence signal of PCC1 was distributed throughout RBCs, suggesting an intracellular distribution (nuclear staining was not observed in RBCs as these cells lack intracellular organelles; Figure 3F). Notably, this fluorescence signal was linearly correlated to the concentration of PCC-1 used during incubation, as observed with CHO-K1 (Figure 3B,G). In addition to entering RBCs, PCC-1 was also hemolytic at high concentrations (Figure S6). Overall, we conclude that PCC-1 can cross the plasma membrane of human cells directly without the requirement of endocytic uptake.

### PCC-1 Disassembly inside Cells

**Figure 4:**
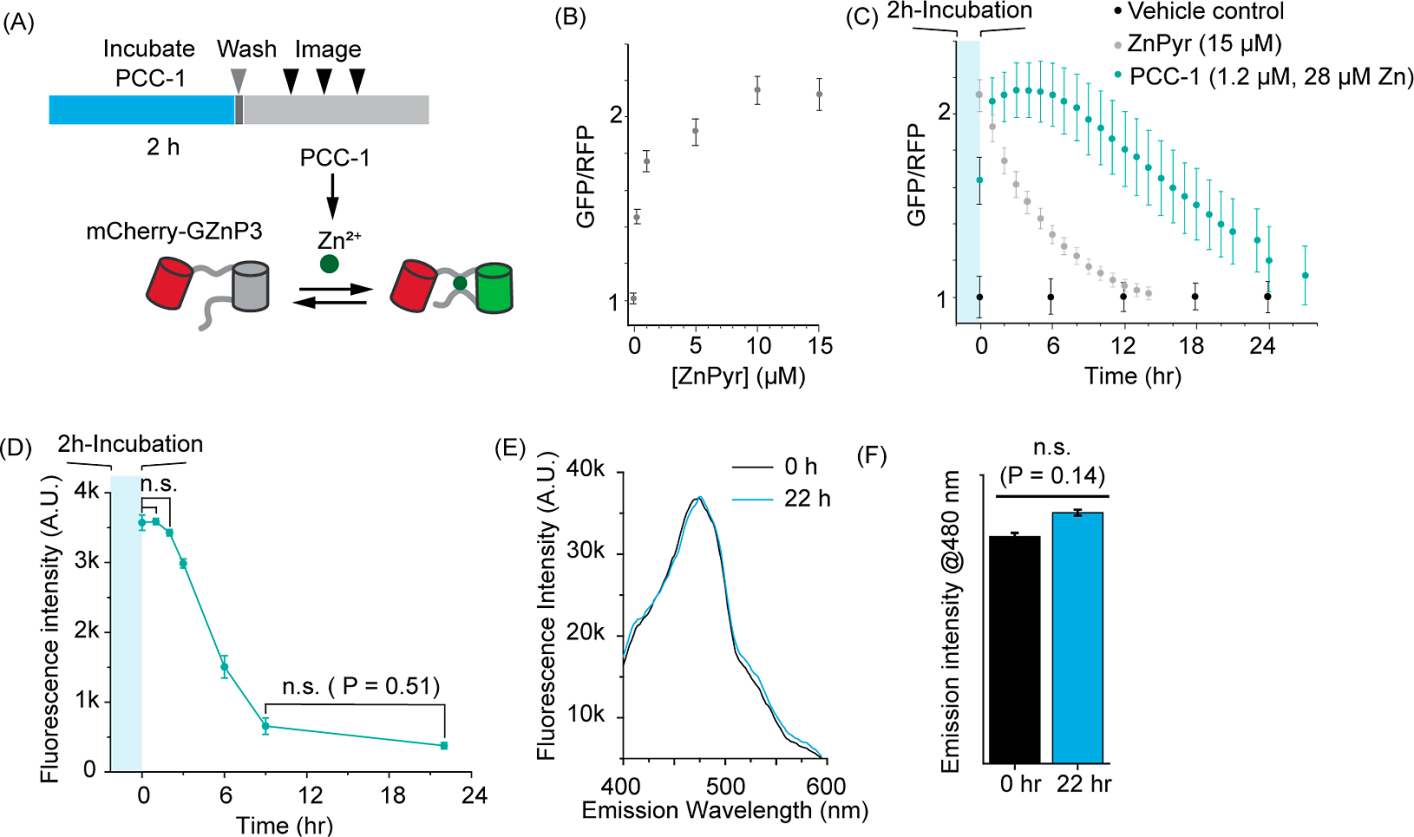
Intracellular disassembly and cellular clearance of PCC-1. (A) Schematic representation of the protocol used to probe PCC-1 disassembly with mCherry-GZnP3. (B) Correlation between the green and red fluorescence signals of mCherry-GZnP3 (GFP/RFP) and the concentration of ZnPyr administered extracellularly. (C) Time-dependent response of the GFP/RFP signal after incubation of PCC-1. Incubations with ZnPyr (15 μM) and vehicle are used as controls. The data represent the average and corresponding standard deviations of biological triplicates, the GFP/RFP signal of approximately 5 × 10^3^ cells being quantified for each condition. (D) Time-dependent response of intracellular PCC-1/H_3_PTH fluorescence after a 2 h incubation of CHO-K1 with PCC-1. The data represent the average intensities and corresponding standard deviations of biological triplicates, approximately 5 × 10^3^ cells being analyzed by experiment. The significance level was evaluated by two-tailed *t* tests (n.s., *P* > 0.05). (E) Fluorescence emission spectra of CHO-K1 cell lysates (cells + media) 0 and 22 h after incubation with PCC-1. (F) Fluorescence intensities at 480 nm of cell lysates of CHO-K1 cells at 0 and 22 h after incubation with PCC-1. The data represent the average intensities and corresponding standard deviations of biological triplicates. (n.s., *p* > 0.05).

To characterize the biocompatibility of PCC-1, we next focused on evaluating the disassembly of this system. Our rationale for this was that the toxicity of PCC-1 may depend on the timing with which the nanocage disassembles into its components. Based on the well-known toxicity of metals,^40^ we were especially interested in monitoring the release of zinc from PCC-1. The release of Zn^2+^ upon intracellular PCC-1 decomposition was quantified by using mCherry-GZnP3, a ratiometric Zn^2+^ sensor. GZnP3, previously described by Qin and co-workers, is a turn-on sensor that detects Zn^2+^ in cells on a second timescale.^51^ GZnP3 is therefore adapted to measure the release of Zn^2+^ from PCC-1 in real time (Figure 4A). To monitor transfection into live cells and quantify the expression levels over time, GZnP3 was fused to red fluorescent protein mCherry (Figure 4A). CHO-K1 cells were first transfected with the mCherry-GZnP3-encoding plasmid (Figure S7). Cells were then cultured for 24 h to allow the expression of the Zn^2+^ sensor. In control experiments, cells were treated for 10 min with different concentrations of zinc pyrithione (ZnPyr), a cell-penetrating coordination complex of zinc (Figure S8). The GFP/RFP ratio obtained from the mCherry-GZnP3 probe correlates with increasing ZnPyr concentration, with an apparent saturation of the probe reached between 5 and 10 μM (the GFP/RFP ratio is constant after 5 min of incubation, indicative of rapid equilibration of intracellular zinc concentrations) (Figure 4B). Consistent with the notion that zinc is exported from cells by zinc transporters,^52–55^ the GFP/RFP ratio diminished to baseline for 12 h upon cell washing (Figure 4C). In comparison, incubation of cells with PCC-1 for 2 h led to a GFP/RFP ratio increasing immediately after incubation and at mCherry-GZnP3 saturation for 6–9 h. As with Zn ZnPyr, the GFP/RFP signal declined to a baseline over time. However, unlike ZnPyr, this response was significantly delayed, requiring more than 24 h of incubation. Together, these results suggest an early phase of PCC-1 decomposition, where the rate of zinc release exceeds that of zinc export. After approximately 9 h, export likely dominates and zinc concentration diminishes.

To probe the fate of PCC-1 inside cells further, the intracellular fluorescence of PCC-1 and its decomposed H_3_PTH ligand was monitored by flow cytometry over the same time course. As observed with zinc release, PCC-1/H_3_PTH fluorescence declined for 9 h (Figure 4D). In parallel experiments, the overall fluorescence of PCC-1/H_3_PTH from samples (lysed cells and media) was quantified directly postincubation and after 22 h (Figure 4D–F). This analysis established that the total fluorescence of the samples is unchanged, indicating that the fluorescent moiety, H_3_PTH, is not catabolized by cells during this incubation period. Instead, these results suggest that H_3_PTH, either in its free ligand form or as PCC-1, is exported out of cells. Overall, the Zn^2+^ and fluorescence detection analyses indicate that PCC-1 and its decomposed components, Zn^2+^ and H_3_PTH, are depleted from cells 24 h postincubation (lacking detection techniques for the phenolate-based vertex H_4_TBSC, the fate of this ligand is unclear).

### Toxicity and Cell Cycle Disruption by PCC-1

**Figure 5:**
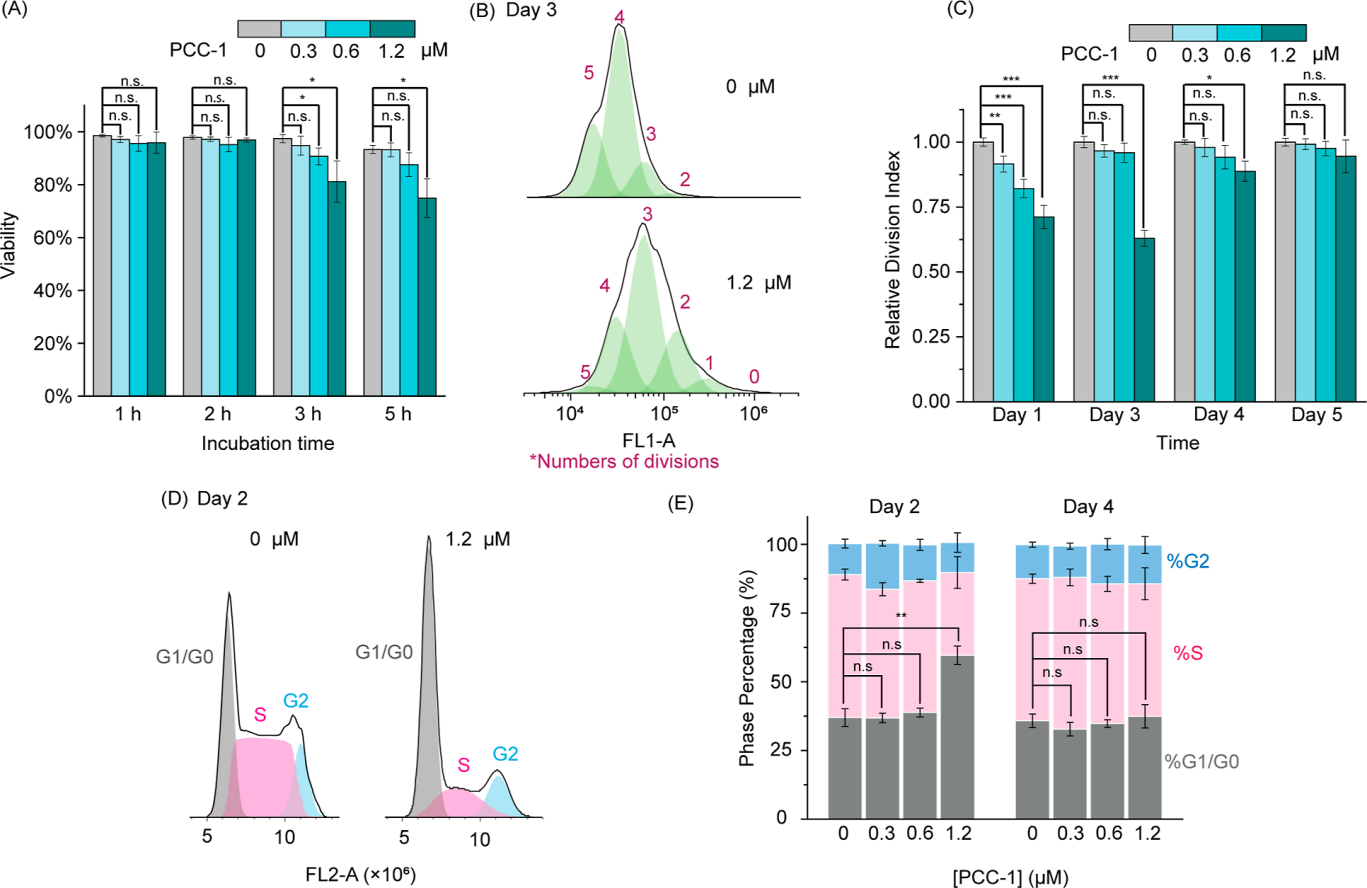
Toxicity and cell cycle disruption effect of PCC-1. (A) Cell viability of CHO-K1 at 24 h after the treatment with PCC-1 at concentrations of 0.3, 0.6, and 1.2 μM, for 1–5 h. The data represent the average intensities and corresponding standard deviations of biological triplicates. The significance level was evaluated by two-tailed *t* tests (n.s., *p* > 0.05; *, 0.05 ≥ *p* > 0.01; **, 0.01 ≥ *p* > 0.001; ***,0.001 ≥ *p* > 0.0001; ****p < 0.001). (B) Representative flow cytometry data obtained from CFSE dilution assay and processed with the FlowJo software. The CFSE fluorescence peaks (black curve) were deconvoluted into multiple peaks (green-shaded peaks) representing cells that underwent different numbers of division, as indicated by the number above each peak. (C) Division index at days 1 through 5 after PCC-1 treatments (Day 0 is the day of PCC-1 treatment). The indexes among different PCC-1 concentration treatments were normalized to the untreated control group at each time point. (D) Representative data of cell cycle analysis assay processed with the FlowJo software. The flow cytometry profile (FL2-A, black line) is proportional to DNA content, and modeled into three different mitotic phases (pseudocolored blue, pink, and gray for G2, S, and G1/G0, respectively). (E) Cell cycle analysis results of CHO-K1 cells treated with PCC-1. The data represent the percentages of cells in G2, S, and G1/G0 phases, at 2 and 4 days after PCC-1 treatments. The data represent the average percentages and corresponding standard deviations of biological triplicates.

Having established relative time windows for cell penetration, clearance, and decomposition of PCC-1, we next investigated the potentially deleterious impact that this nanocage may have on cells. The toxicity of PCC-1 on CHO-K1 cells was assessed by quantifying the percentage of cells stained with SYTO 59 and SYTOX Green by flow cytometry, SYTO 59 staining all cells, and SYTOX Green staining dead cells with compromised plasma membranes (Figure S9). Cells were treated with PCC-1 (0, 0.3, 0.6, or 1.2 μM) for 1–5 h, washed, and incubated in growth media for 24 h. The viability of the CHO-K1 cells was generally unaffected by these treatments, except for the highest concentration condition tested (Figure 5A). Indeed, 1.2 μM PCC-1 reduced the viability to approximately 80 and 75% when incubated for 3 or 5 h, respectively. Overall, these results indicated that a 2 h treatment is not toxic to cells. To investigate whether PCC-1 may lead to potentially deleterious effects of PCC-1 on cells under these conditions, cellular proliferation rates were monitored after the PCC-1 treatment as a proxy for overall metabolic health. We used the carboxyfluorescein diacetate succinimidyl ester (CFSE) dilution assay for this analysis.^56^ In this assay, cytosolic proteins are covalently labeled with fluorescein, and each cell division leads to a dilution of this signal that can be accessed by flow cytometry. CFSE-labeled cells were treated with PCC-1 (0, 0.3, 0.6, or 1.2 μM) for 2 h and maintained in standard growth media. The intracellular fluorescence was analyzed by a flow cytometer for 5 days. This time frame was chosen because it encompasses the 24 h required for PCC-1 clearance and decomposition (as determined in the prior section) and several additional days likely necessary to monitor potential recovery. Proliferation parameters were calculated from the flow cytometry analysis performed on individual days using FlowJo Software^57^ (Figure 5B). Based on the division index, which represents the average number of cell divisions undergone compared to a control with no treatment, PCC-1 reduced the proliferation rate at day 1 in a manner proportional to concentration (Figure 5C). Notably, all concentrations impacted cell proliferation, even those that did not contribute to cell toxicity (0.3 and 0.6 μM). The division index for the 0.3 and 0.6 μM conditions returned to the control level on day 3. The division index for the 1.2 μM conditions was still diminished at this time, indicating that cells are slower to recover from this higher-concentration treatment. Likewise, this effect persisted until day 4. However, at day 5, all cells proliferated at similar rates, indicative of a return to homeostasis for all samples. Cell cycle analyses were performed to confirm these results further.^58–60^ Cellular DNA staining with a fluorescent dye and flow cytometry were used to quantify cells in the G0/G1, S, or G2 phases (Figure 5D). The phase percentages of cells treated with 0.3 or 0.6 μM PCC-1 were indistinguishable from those of untreated cells on day 2, indicating that the proliferation profiles of these cells are identical (Figure 5E). This is in agreement with the division index measurement. In contrast, approximately 60% of cells treated with 1.2 μM PCC-1 were in the G0/G1 phase on day 2, as opposed to approximately 37% for the control group. Hence, many cells are in cell cycle arrest for this condition, consistent with a reduced cell division index. Nonetheless, on day 4, the percentages of G0/G1, S, and G2 phases in the PCC-1 treatment groups matched those in the control group, indicating that this time frame is sufficient for cell recovery.

## Discussion

Nanocarriers, including liposomes,^19,61–63^ metal/metal oxide nanoparticles,^64–66^ polymer nanoparticles,^67–69^ and nanosized metal–organic frameworks,^70–72^ are usually relatively large, with diameters spanning tens of nanometers. As a result, nanocarriers are typically unable to cross membranes, and they are generally internalized into cells by endocytic uptake mechanisms.^15,20^ Notably, this is the case for other reported PCCs. For instance, the PCCs, ZnPMTC,^31^ and Zn-NH-pyr,^30^ reported by Zhang, Dai, and co-workers were internalized into cells through endocytosis, as evidenced by a punctate intracellular fluorescence and colocalization with endosomal/lysosomal markers. In contrast, our results showed that PCC-1 localizes in the nuclei and cytoplasm without apparent endosomal accumulation, as indicated by the absence of fluorescent puncta. This phenomenon is also independent of sodium azide, a metabolic inhibitor that blocks energy-dependent processes, including endocytosis.^46–48^ Cell penetration is also detectable in erythrocytes, cells devoid of endocytic uptake, or membranous organelles. In turn, this points to a direct plasma membrane translocation model. This conclusion is surprising as membrane permeability is limited for molecules with volumes of 1500 Å^3^.^73,74^ Indeed, some of the largest molecules reported to cross cell membranes are cyclosporin A (MW 1.2 kDa, 1400 Å^3^)^75^ or TAT-like cell-penetrating peptides (∼1.5 kDa).^76^ At an expected 12 kDa and 70,000 Å^3^, PCC-1 is far above this limit.

**Figure 6:**
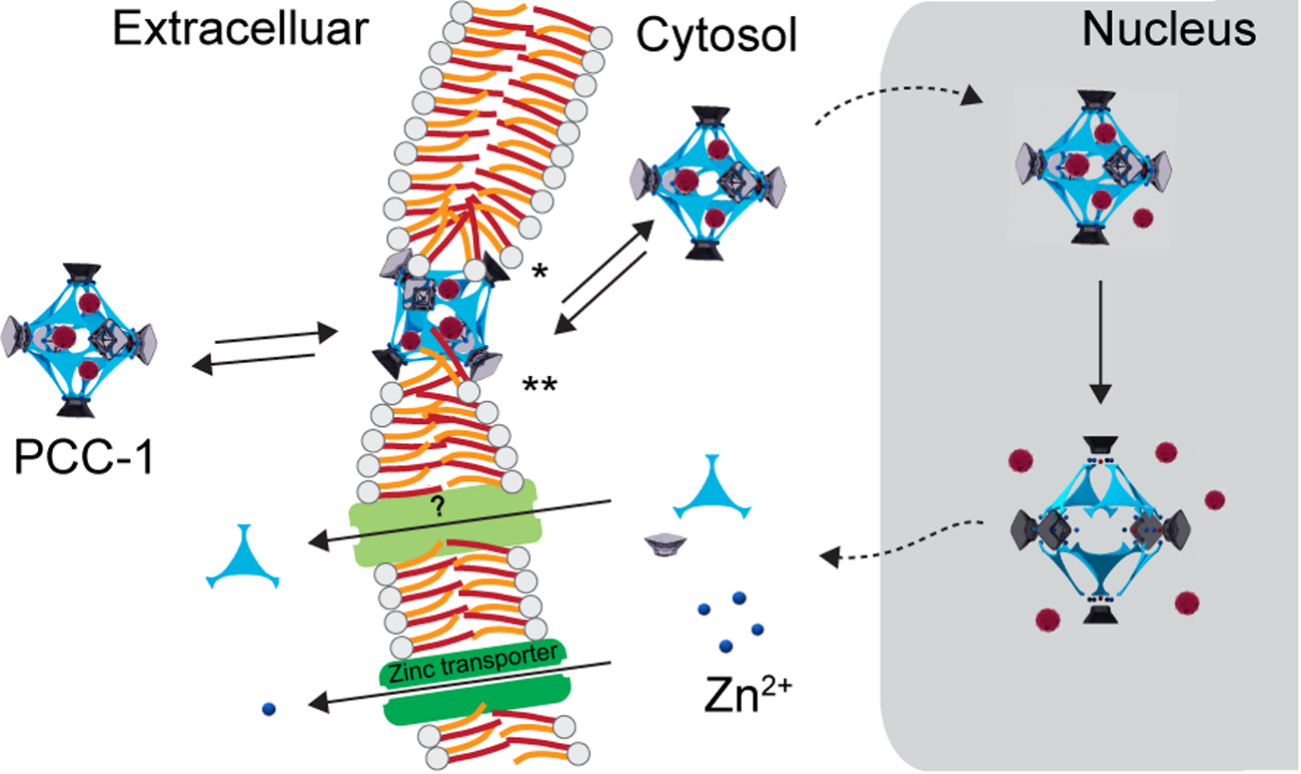
Model of the transport of PCC-1 and its decomposed components in and out of human cells. The plasma membrane translocation step is shown with two potential scenarios: (a) interactions with lipid polar heads (highlighted by an asterisk) and (b) interactions with the hydrophobic region of the bilayer (highlighted by a double asterisk). The diameter of PCC-1 approximates the width of a lipid bilayer, which is around 4 nm. Once in the cytosol, PCC-1 targets the cell’s nucleus, associating with both nuclear and nucleolar factors. This likely causes retention of the nanocage in this organelle and slows down its export. The guest payload may be released from PCC-1 both while the nanocage remains intact and as it degrades. Inside the cell, PCC-1 exhibits potential cytotoxic effects. However, below a threshold concentration, the nanocage imparts only a temporary impact on the cell cycle and on proliferation. Notably, cells demonstrate a rapid recovery, a process that aligns with the expulsion of PCC-1 and its disintegrated elements.

Several scenarios may explain the direct plasma membrane translocation of a structure, such as PCC-1. First, it is possible that PCC-1 does not cross the membrane in the fully extended crystal structure described in Figure 1. Instead, PCC-1 could partially collapse before transport to present a small surface area (a model proposed for cyclosporin A^77^). However, the ability of PCC-1 to transport MB into cells disagrees with this idea, as the loading of the endo cavity would prevent this collapse. A counterargument is that MB may associate with the surface of PCC-1, not with its core. This surface association would likely increase the size of the nanocarrier, change its interaction with cellular components, and impact intracellular transport rate or efficiency. However, PCC-1 and MB@PCC-1 show similar behavior with regard to their cell penetration. Hence, endo encapsulation is more plausible than exo encapsulation. Another scenario that could account for the membrane translocation of PCC-1 involves the involvement of a protein transporter. The linear relationship between the transport rate detected for PCC-1 and its extracellular concentration is consistent with a passive simple diffusion model. However, the transport of PCC-1 may be carrier-mediated, assuming a protein transporter with a saturation threshold far above the concentration used herein (cytotoxicity becoming a problem at high concentrations). Possible clues in this context are that PCC-1 is hemolytic at high PCC-1/RBC ratios and that PCC-1 can cause shrinkage of RBC ghosts. Above a certain threshold, PCC-1 can therefore permeabilize membranes and extract membrane components from bilayers. Hence, PCC-1 may have a detergent-like character. This detergent-like property could account for PCC-1 translocating across a lipid bilayer passively without help from protein transporters. We provide two possibilities by which this could happen in Figure 6. In one scenario, lipid polar heads could surround PCC-1 and favor the formation of inverted micelles. In a second scenario, the hydrophobic surface of the nanocage may simply embed into the hydrophobic fatty acid alkyl chains. In both cases, transport is driven by concentration gradients. Biophysical studies between PCC-1 and the bilayers should help test the validity of these models in the future.

The future utility of PCCs will likely be related to their ability to deliver drugs without causing undesirable toxicities. In the case of PCC-1, each nanocage potentially brings 24 zinc atoms, six vertex, and eight ligands into cells, assuming complete decomposition. Zinc is an essential trace element that plays a vital role in various biological processes such as cell division,^52^ immune function,^41^ and protein synthesis.^78^ However, excessive concentrations will disrupt homeostasis^79^ and potentially lead to oxidative stress,^80^ disruption of cellular signaling,^81^ and inhibition of enzyme activity:^82^ Excess zinc can also compete with other essential trace elements, such as iron and copper, for binding sites on transport proteins and enzymes.^83–85^ On the one hand, prior studies have established that zinc supplementation is relatively safe and even beneficial in treating various conditions.^41^ On the other hand, various cell culture assays, animal studies, and human case reports have highlighted the potential dangers of high zinc concentrations. With this in mind, one of our goals was to investigate the interplay among PPC-1 cell entry, its decomposition in live cells, the subsequent release of zinc, and finally, its cellular effects. We found PCC-1 to be toxic to CHO-K1 at 1.2 μM. This toxicity was avoided at lower concentrations and if the incubation time was limited. This suggests that the nanocage does not immediately damage cells upon contact or via the cell penetration process itself. Notably, because the 3 and 5 h incubation times do not lead to intracellular PCC-1 concentrations higher than 2 h, it is unlikely that toxicity arises by the accumulation of PCC-1 itself. Instead, a zinc reporter probe indicated that PCC-1 releases zinc upon cell entry on a timescale of several hours. The precise amount of zinc released is hard to predict and calibrate. Our current results indicate that 1.2 μM PCC-1 released at least as much zinc as is introduced into cells by a treatment of 10 μM ZnPyr (approximate saturation threshold of the reporter). This zinc release also coincided with the export of the fluorescent ligand from cells. Together, these results suggest that a population of PCC-1 decomposes into its components and that the cells can export these components (Figure 6). The export of excess zinc is expected based on the well-characterized existence of zinc transporters. The mechanism of export for the ligand (which would be present in an amount proportional to the zinc released in a 1:3 ratio) is currently unclear. However, because the H_3_PTH ligand does not enter cells, it likely cannot cross membranes by diffusion. Other mechanisms are possible, including export by ABC transporters (exporters that remove waste products and xenobiotics from cells).^86^ Of course, the export of intact PCC-1 may also contribute to the loss of fluorescence observed over time. In this context, it is worth noting that while it takes 2 h for PCC-1’s fluorescence to reach a steady state in cells, fluorescence intensities inside cells do not substantially change for at least 2 h postwashing. PCC-1 is retained inside cells instead of being immediately exported, presumably because of the strong association with the nucleus and nucleolus observed in permeabilized cells. Overall, PCC-1 and its components appear to be cleared out of cells in approximately 24 h (for 1.2 μM, 2 h incubation). Notably, lingering effects on the cell cycle can be detected beyond this 24 h period. Indeed, cell proliferation is perturbed under these conditions for at least two additional days. Overall, these studies of PCC-1 show that PCC-1 can kill cells and, at sublethal doses, cause temporary inhibition of the proliferation of CHO-K1 cells. However, controlling the concentration and incubation time can avoid these deleterious effects. Moreover, cells can recover and return to apparent homeostasis within a time window of a few days.

Prior reports have established that PCC-1 could transport the hydrophobic drug CPT directly into the nucleus of cancer cells, in tissue cultures, and in a xenograft mouse model.^26^ The goal of these studies was to test whether CPT@PCC-would kill cancer cells or reduce their proliferation better than CPT alone. It is interesting to note that, while the potency of CPT@PCC-1 is attributable to the encapsulation and release of CPT by PCC-1, PCC-1 itself may have provided an antiproliferative effect and potentially acted in synergy with the drug. Our current results also highlight how PCC-1 may be useful for therapeutic applications not involving cell killing. Indeed, there appears to be a concentration window in which PCC-1 does not drastically impact cell homeostasis. What concentrations are tolerable and useful in vivo remains to be determined. Whether PCC-1 can be applied to nonkilling therapeutic approaches also remains to be established. In principle, our results indicate that this is possible, but it would likely require drugs that can work at low intracellular concentrations, so as to minimize the dose of PCC-1 required. The ability of PCC-1 to cross membranes directly is likely to intimately control the in vivo pharmacokinetics and pharmacodynamics of this potential drug delivery system. In this regard, future studies that compare PCC-1 to PCCs prone to endocytosis should help to establish the strengths and limitations of each respective drug delivery platform. The cellular characterization provided herein will provide guiding principles for such in vivo studies.

## Supporting Information Available

Material and methods, synthesis protocol of PCC-1, cargo molecule loading protocols, characterization of PCC-1 decomposition in vitro, cell culture protocols, PCC-1 treatment and imaging, quantification of the cellular uptake of PCC-1 and H3PTH ligand by flow cytometry, quantification of the delivery of propidium iodide mediated by PCC by flow cytometry, PCC-1 uptake in red blood cells, PCC-1 decomposition assay in live cells, PCC-1 uptake and export experiments, toxicity assay, cell proliferation assay, cell cycle analysis, absorbance and fluorescence analysis of H3PTH, PCC-1, and MB@PCC-1, and NMR spectroscopy analysis of MB encapsulation by PCC-1 (PDF)
