# Supplementary material for: Investigating the Cell Entry Mechanism, Disassembly, and Toxicity of the Nanocage PCC-1: Insights into Its Potential as a Drug Delivery Vehicle
Source: J Am Chem Soc. 2023 Dec 9;145(50):27690–701. doi: 10.1021/jacs.3c09918 (PMC10863074; doi:10.1021/jacs.3c09918)
Supplement: Supplementary file 1 — ja3c09918_si_001.pdf [file ja3c09918_si_001.pdf]

Supporting Information

Investigating the cell entry mechanism, disassembly, and toxicity of the nanocage PCC-1: insights into its potential as a drug delivery vehicle.

Zhifeng Xiao[2], Hengyu Lin[2], Hannah F. Drake[2], Joshua Diaz[1], Hong-Cai Zhou[2] & Jean-Philippe Pellois[1,2*]

From [1]Department of Biochemistry and Biophysics, Texas A&M University, College Station, TX 77843, USA;

[2]Department of Chemistry, Texas A&M University, College Station, TX 77843, USA.

*To whom correspondence should be addressed

Address correspondence to: Jean-Philippe Pellois, Biochemistry and Biophysics Bldg., Room 430, 300 Olsen Blvd, College Station, TX, 77843-2128.

Fax: 979-862-4718, E-mail: pellois@tamu.edu

Orcid ID: Jean Philippe Pellois (0000-0001-8528-4652)

Contents

Reagents 2

Synthesis of PCC-1 2

Cargo molecule loading experiments 4

PCC-1 decomposition in vitro. 5

Cell cultures 6

PCC-1 treatment and Imaging 6

Cellular uptake of PCC-1 vs H3PTH ligand, as quantified by flow cytometry....................7

Delivery of propidium iodide mediated by PCC-1, as quantified by flow cytometry..........8

PCC-1 Uptake in Red Blood Cells 8

PCC-1 decomposition assay in live cells 9

PCC-1 uptake and export experiments 11
Cell Proliferation Assay..13
Cell Cycle analysis..15
Absorbance and Fluorescence Analysis of H3PTH, PCC-1 and MB@PCC-1 16
NMR spectroscopy analysis of MB encapsulation by PCC-1 17
References 19

Reagents

Histone H1 From Calf Thymus, Alexa Fluor™ 488 Conjugate (Invitrogen), SYTOX Green (5 mM Solution in DMSO, Invitrogen), SYTO 59(5 mM Solution in DMSO, Invitrogen), methylene blue (Sigma-Aldrich), Quinine sulfate dihydrate (Thermo Scientific Chemicals), H 1152 dihydrochloride (Sigma-Aldrich), Propidium iodide (Sigma-Aldrich), Rhodamine B (Sigma-Aldrich), Rhodamine B base (Sigma-Aldrich), Rhodamine 6g (Sigma-Aldrich), bezafibrate(Selleck), retinoic acid (TCI Chemical, Resveratrol (Thermo Scientific Chemicals), fluorescein (Thermo Scientific Chemicals), fluorescein disodium salt (Thermo Scientific Chemicals), Erythrosin B sodium salt (Sigma-Aldrich), N-phenyl phenothiazine (Ambeed), RNase A (Thermo Scientific), Digitonin (ACROS Organics), Zinc pyrithione (ALFA Chemistry), Lipofectamine 3000 (Invitrogen).

Synthesis of PCC-1

The synthesis of H_3PTH was following the same protocol reported previously[1]. The synthetic scheme is shown in Figure S1.

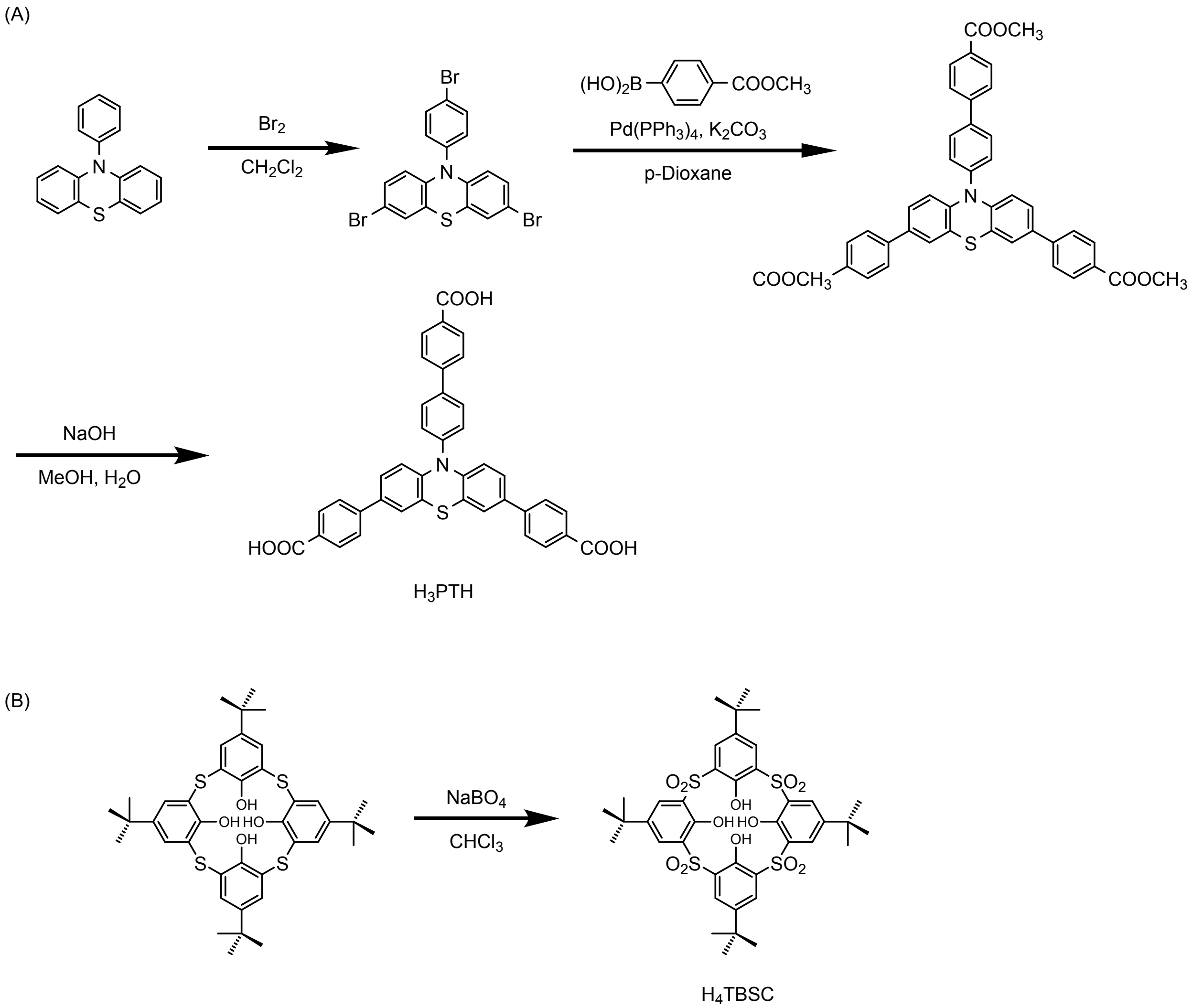

Figure S1. Synthetic Schemes of PCC-1 ligands (A) H_3PTH;(B) H_4TBSC

The synthetic protocols for formation of PCC-1 crystals were adapted from previously reported procedures[1] to provide higher yields. Dry $ZnCl_2$ (60 mg), H_3PTH (25 mg) and H_4TBSC (15 mg) are combined in a 4 mL glass vial. DMF (1 mL) is added to the vial, the vial is capped, and the mixture is sonicated until complete dissolution. The solution is left in a 135 °C oven for 2 days. The solution, which contains visible crystals, is allowed to cool down at room temperature for one day. The crystals of PCC-1 are separated from the solution by centrifugation and decanting. To remove unreacted ligands and metal salts, the crystals are washed with DMF (2 X 1 mL) and acetone (2 X 1 mL). The PCC-1 crystals are then incubated in dry acetone for 3 days for solvent

exchange, dry acetone being replaced daily. PCC-1 crystals are then washed with supercritical CO_2 to remove solvent from pores. The final yield is 10 mg of solvent-free PCC-1.

Cargo molecule loading experiments

A cargo molecule is dissolved in acetonitrile (1 mL, 0.6 mM, 0.6 μmol) and mixed with activated PCC-1 crystals (1 – 2 mg, 0.08-0.12 μmol). The suspension is shaken at room temperature until saturation. The concentration of the cargo molecules remaining in the supernatant is determined by HPLC using a standard curve after dilution.

Cargo molecules that are not soluble in acetonitrile are dissolved in DMSO instead (6 mM). This stock solution is diluted 10 times into acetonitrile to make a 10% DMSO working solution for encapsulation experiments.

The encapsulation ratio (n, number of cargos per PCC-1 cage) was determined using Equation 1. In the equation, c_i is the initial concentration of cargo molecules and c_e is the concentration of cargo remaining in solution after the encapsulation. V is the volume of the cargo molecule concentration. The m_{PCC-1} and M_{PCC-1} are mass and molar mass of PCC-1. The encapsulation reaches equilibrium in 1-2 days for all the tested molecules. Encapsulation kinetics of methylene blue, rhodamine 6g and retinoic acid are shown in Figure S2.

$$n = \frac{(c_i - c_e) \times V}{m_{\mathrm{PCC-1}}/M_{\mathrm{PCC-1}}} \qquad \mathbf{1}$$

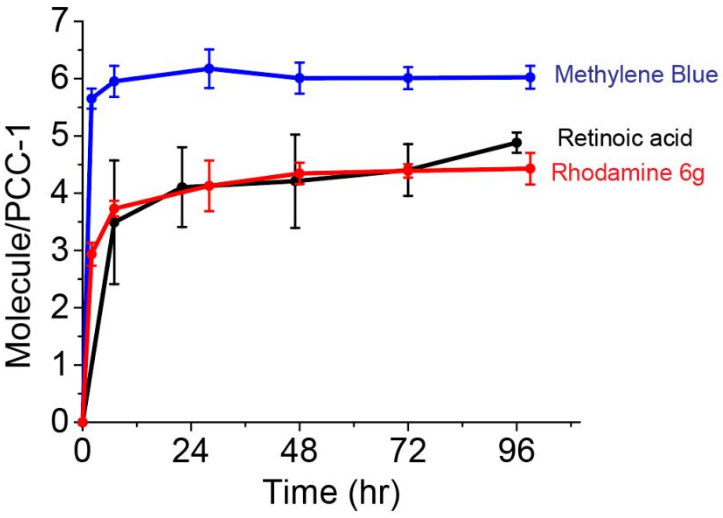

Figure S2. Adsorption kinetics of representative molecules

Calculator Plugins were used for Log D prediction and calculation, ChemAxon (http://www.chemaxon.com). The fitting between Log *D* and cargo/PCC-1 among neutral and anionic cargoes is conducted by Origin Pro™ using the Two-Parameter Exponential Function. The fitting is converged at a χ^2 tolerance value of 10^{-9}. The COD (R^2) of the fitting is 0.53 and the reduced χ^2 is 1.41.

PCC-1 decomposition in vitro.

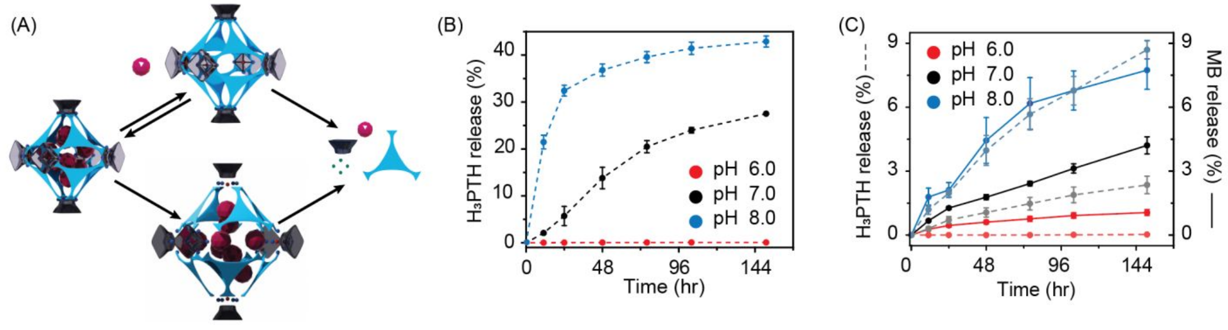

Figure S3. (A) The release of guest molecules from PCC-1 cages can follow either of the pathways. The guest molecule can diffuse out of the cavity followed by hydrolytic decomposition of PCC-1. Alternatively, PCC-1 can decompose and release the encapsulated guests. The two release pathways can be distinguished and quantified by relative guest release rates and PCC-1 decomposition rates. (B)&(C) Aqueous stability and guest release studies on PCC-1 under different pHs, using MB as a guest. In the

experiments, the solids of PCC-1 and MB@PCC-1 were submerged into PBS with different pHs, vortexed and sampled at different time points. At each time point, the mixture was spun down and the supernatant was taken for quantification of MB and H_3PTH concentration with HPLC and the supernatant was replaced with fresh PBS to avoid saturation. (B) The release of H_3PTH was exploited to quantify the hydrolytic decomposition of PCC-1 in aqueous solution. Faster release of H_3PTH from PCC-1 was observed under higher pHs. (C) release rates of MB follow the same trend as the hydrolytic decomposition rate of PCC-1 under different pHs. At pH 8, the release of MB matches the hydrolytic decomposition of PCC-1. At pH 6, the release of MB is slower and independent of PCC-1 decomposition. At pH 7, the processes of decomposition and escape, as described in A, appear to co-exist.

Cell cultures

CHO-K1 cells were maintained in Ham's F-12K (Kaighn's) Medium supplemented with 10% fetal bovine serum (FBS) and 50 U/mL Penicillin-Streptomycin at 37 °C and 5% CO_2. Subculturing was conducted every 2 days in splitting ratios of 1:6 to 1:10.

PCC-1 treatment and Imaging

Activated PCC-1 crystals were dissolved in sterilized DMSO (2.8 mg/mL) by sonication for an hour. After sonication, the undissolved solids are removed by centrifugation (5 min, 14000 rpm). The resulting concentration (0.12 mM) of this PCC-1 stock solution is determined by HPLC, using a standard curve of H_3PTH. To treat the cells with PCC-1, the stock solution is diluted into Leibovitz's L-15 Medium in a stepwise manner. For example, to prepare 1.0 mL of PCC-1 treatment solution (1.2 μM), 10 μL of the stock solution is mixed with 90 μL of L-15, and then further diluted with 900 μL of L-15. After treatment, cells are washed with PBS (3 X) and incubated in L-15 during fluorescence microscopy imaging.

To observe the localization of PCC-1 and H_3PTH in permeabilized cells, the cells are first treated with digitonin. A digitonin stock solution (20 mg/mL) is prepared in DMSO. This digitonin stock solution is diluted in PBS to a working concentration of 0.1 mg/mL. To permeabilize cells in an 8-well plate (Lab-Tek II), the cells are washed with cold PBS (3 X), incubated with cold PBS (0.2 mL), and placed on ice. The digitonin solution (0.2 mL of 0.2 mg/mL) is added to the well. The cells are incubated on ice for 5 min and the

solution is removed by aspiration. The cells are washed with PBS (1 X, room temperature), and treated with PCC-1 (1.2 μM in L15) for an hour. Cells are washed with PBS (3 X) and incubated in L-15 for imaging.

To stain cells with SYTOX AADvanced, the imaging media is supplemented with SYTOX AADvanced (1 μM, from a DMSO stock solution). Cells are then incubated at 37 °C for 10 min before imaging. Fluorescence microscopy images were acquired using the 100X objective on an Olympus IX-81 microscope.

Cellular uptake of PCC-1 vs H3PTH ligand, as quantified by flow cytometry

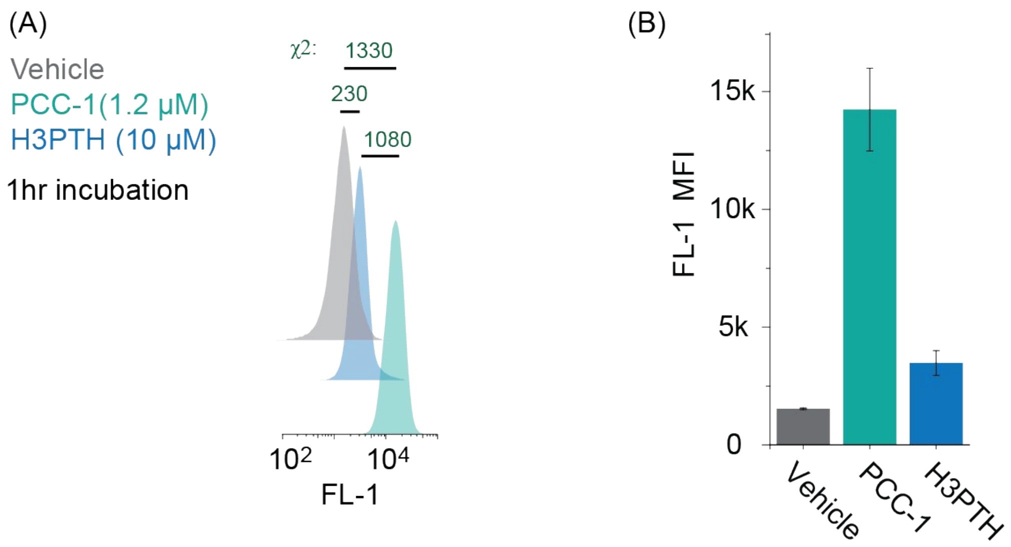

Figure S4. Cellular uptake studies of H3PTH characterized by flow cytometry. CHO-K1 cells were treated with H3PTH for one hour. The vehicle is DMSO. (A) Comparison of intracellular fluorescence (FL-1 channel) among different treatments. (B) Comparison of the mean fluorescence intensity (MFI) of the cell population, as analyzed using the FlowJo software. The fluorescence intensities reported correspond the MFI obtained of cell populations analyzed by flow cytometry. The data represent the average MFI and corresponding standard deviations of biological triplicates. Each replicate uses 4×10^4 cells for analysis.

Delivery of propidium iodide mediated by PCC-1, as quantified by flow cytometry.

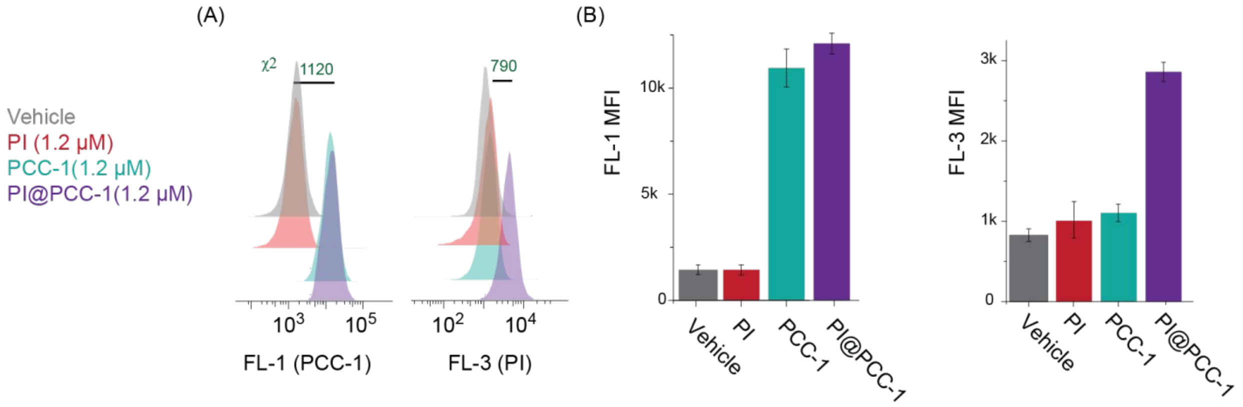

Figure S5. PI delivery studies characterized by flow cytometry. CHO-K1 cells were treated with dissolved PI@PCC-1 for one hour and analyzed with flow cytometry. FL-1 represents the intracellular fluorescence from PCC-1, while FL-3 represents the fluorescence from PI. (A) Comparison of intracellular PCC-1 fluorescence (FL-1 channel) and PI fluorescence (FL-3 channel) among different treatments. (B) Comparison of the mean fluorescence intensity (MFI) of the cell population, as analyzed using the FlowJo software. The fluorescence intensities reported correspond the MFI obtained of cell populations analyzed by flow cytometry. The data represent the average MFI and corresponding standard deviations of biological triplicates. Each replicate uses 4×10^4 cells for analysis. Note: PI-stained cells were not stained by SYTOX green after treatment with PI@PCC-1, indicating that the cells are not dead.

PCC-1 Uptake in Red Blood Cells

Uptake experiments are also conducted in human Red Blood Cells (RBCs) purchased from the Gulf Blood Bank (Galveston, TX). To separate RBCs from the whole blood samples, the sample is centrifuged for 5 min at 1500g. The RBCs pellet obtained is resuspended in PBS. The same centrifugation and resuspension are conducted three times in PBS to remove the plasma and buffy coat. The RBCs are eventually resuspended in PBS to a 50% suspension as a stock and stored at 4°C. Uptake experiments are conducted in a 200 μL 0.1% RBC L-15 solutions. PCC-1 treatments are introduced into the cells by stepwise dilution into the final volume with L-15.

To image the cells under a fluorescence microscope (Olympus IX-81 microscope, 100X objective), the cells are incubated with PCC-1 for one hour. Then, 50 μL of the treated RBCs solution is centrifuged (5min, 1500g) and resuspended in L-15 (200 μL). BODIPY C11 is introduced into the media by adding its DMSO stock solution (1 mM) to reach a

final concentration of 1 μM. The cells are incubated for 5 min for optimal staining. The RBCs are transferred to an 8-well glass bottom plate (LabTek II) and allowed to settle to the bottom for imaging.

To measure the PCC-1 uptake in RBCs, the intracellular fluorescence is determined by a flow cytometer after one hour of incubation with PCC-1. The data were processed by FlowJo™ software. During data processing, multiplets of RBCs are removed from the analyses. The mean fluorescence of single RBCs is used to correlate the treatment concentration and intracellular PCC-1 concentration.

The hemolysis of RBCs under 1-hour PCC-1 treatments of different concentrations is characterized by the absorbance of hemoglobin leaking out of the cells. Full hemolysis is obtained by incubating the RBCs with 0.1% Triton X-100 PBS solution (pH7.4). The percentage of hemolysis is normalized to full hemolysis (Figure S8).

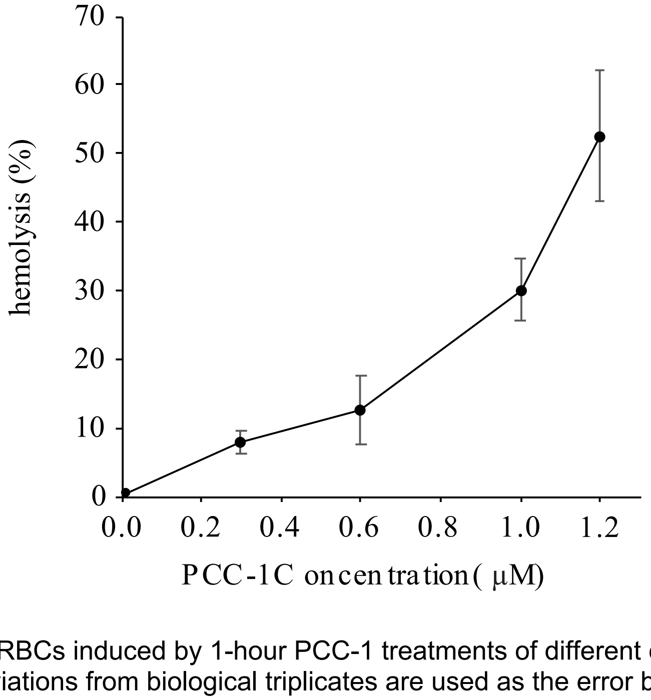

Figure S6. Hemolysis of RBCs induced by 1-hour PCC-1 treatments of different concentrations. Standard deviations from biological triplicates are used as the error bar.

PCC-1 decomposition assay in live cells

The ratiometric intracellular Zn reporter protein, mCherry-GZnP3 was constructed by molecular cloning from plasmids obtained from Addgene (GZnP3 in pcDNA3.1+,

#161738, and pmCherry-Gal3, #85662). The vector backbone containing mCherry sequence was obtained by digestion of pmCherry-Gal3 using XhoI and EcoRI. The DNA sequence encoding GZnP3 was amplified from GZnP3 in pcDNA3.1+ using primers containing 5'XhoI (Primer 1,) and 3'EcoRI sites (Primer 2). The digested PCR product was ligated to the mCherry gene backbone to construct pmCherry-GZnP3.

Primer 1: 5'-atatatCTCGAGctcaagctttggcaatgttcaaacaccctatggaa-3'

Primer 2: 5'-gggccgGAATTcctaatCttgagagacatgatc-3'

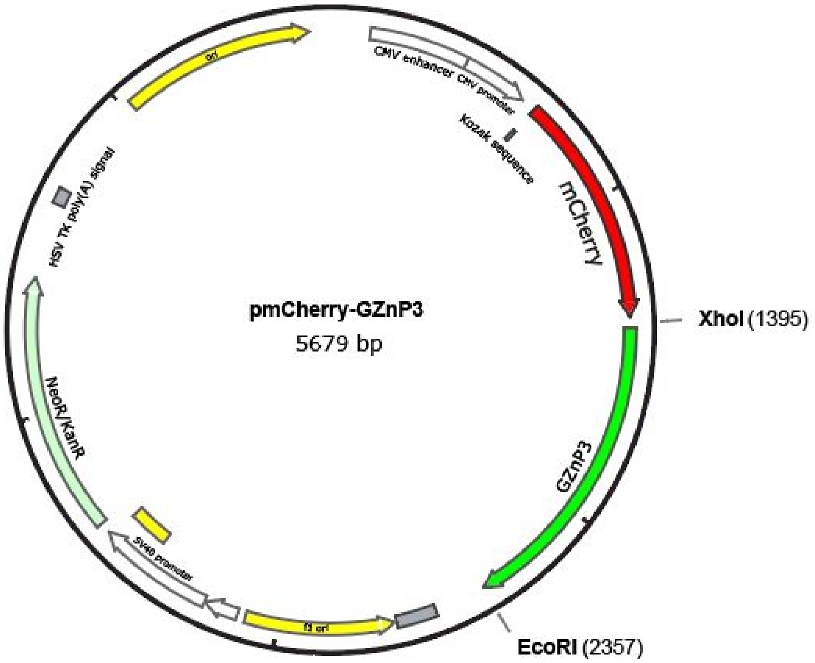

Figure S7. Plasmid map for pmCherry-GZnP3

CHO-K1 cells are transiently transfected with pmCherry-GZnP3 by using Lipofectamine 3000. In a typical transfection experiment in a 48-well plate, pmCherry-GZnP3 DNA (400 ng) and Lipofectamine 3000 (0.6 μL) are mixed. The transfection reagent is incubated with CHO-K1 cells for 24 hours. Cells are washed with PBS (3X) and incubated in fresh media.

A stock solution of Zinc pyrithione (ZnPyr, 5 mM) is prepared in DMSO. In a typical experiment, ZnPyr treatment solution was made by diluting the stock solution into PBS.

Cells are treated with Zn pyrithione (0.1 – 15 µM, 10 min). After the treatment, the cells are washed with PBS (3X) and incubated in the complete growth media. Alternatively, cells are treated with PCC-1 (1.2 µM, 2 h), as described in previous sections.

Cells are imaged using an EVOS FL Auto 2 inverted microscope (Thermo Fisher Scientific). The microscope is equipped with a heating chamber maintained at 37 °C. Images are acquired using bright field imaging and five fluorescence filter sets: CFP (Ex = 436 ± 10 nm, Em = 480 ± 20 nm), RFP (Ex = 560 ± 20 nm, Em = 630 ± 35 nm), GFP (Ex = 488 ± 10 nm Em = 520 ± 20 nm), DAPI (Ex = 350 ± 50 nm, Em = 460 ± 25 nm), Cy5 (Ex = 350 ± 50 nm, Em = 460 ± 25 nm). Fluorescence images are taken every hour for 24 hours and are analyzed by ImageJ program to quantify the ratio of GFP and RFP channels fluorescence intensity.

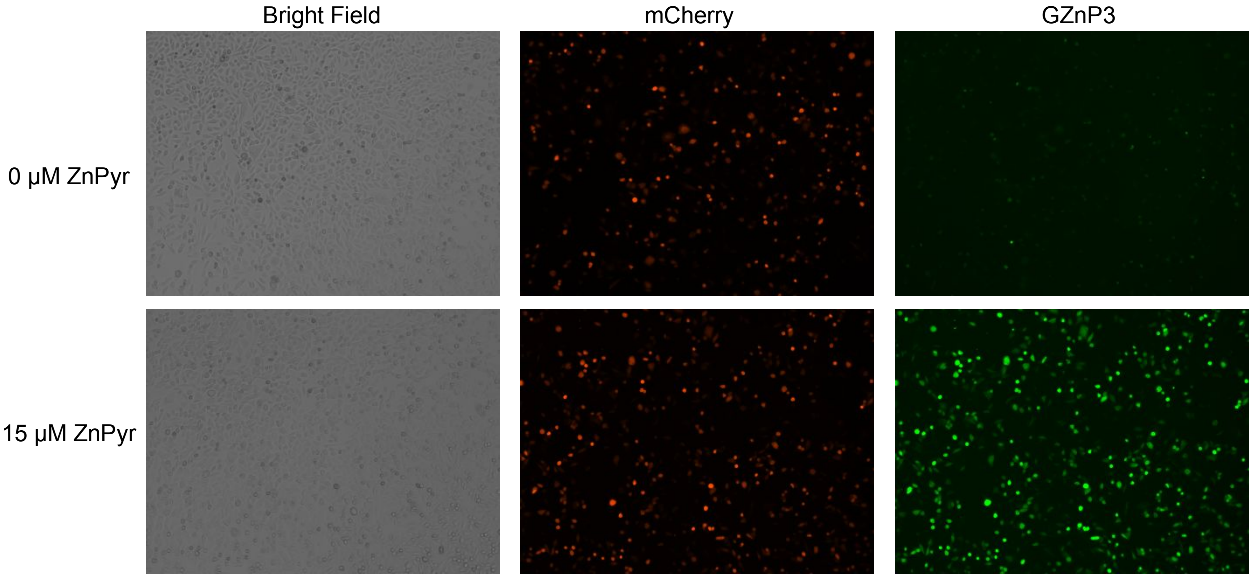

Figure S8. mCherry-GZnP3 response to ZnPyr treatment in transfected CHO-K1 cells

PCC-1 uptake and export experiments

<u>PCC-1 Uptake in CHO-K1 cells</u>

The uptake of PCC-1 into CHO-K1 cells is characterized by the intracellular fluorescence of PCC-1. The CHO-K1 cells are plated into a 48-well plate with the amount of 50k/well one day ahead of the experiment. These cells were treated with different concentrations

of PCC-1 (0.3, 0.6 and 1.2 μM) for different time periods (1, 2, 3, and 5 hr) before they are harvest by trypsinization. The intracellular fluorescence of harvested cells is analyzed by a BD Accuri C6 flow cytometer under the FL-1 channel (Ex 488 nm, Em 533/30 nm). Flow cytometry data were processed using FlowJo™ Software[2].

To obtain ATP-depleted cells, the cells are treated with growth medium supplemented with sodium azide (40 mM) for 5 hours. After the sodium azide treatment, the cells are washed and treated with PCC-1 (0.3, 0.6 and 1.2 μM) or AF488-H1 for an hour. After treatment, the cells are washed by PBS (3X), harvest by trypsinization and analyzed by the flow cytometer.

PCC-1 Export Experiments

The export of PCC-1/H_3PTH after uptake is characterized exploiting the intracellular fluorescence. The cells are first treated with PCC-1 (1.2 μM) for 2 h, then washed with PBS and incubated with complete growth media for different time periods. At each time point, the cells are harvested by trypsinization from the wells and analyzed by the flow cytometer. The total fluorescence of the cell and media are measured with a fluorometer to obtain emission spectra under 360 nm excitation. Before the measurement, the cells are lysed by sonication.

The flow cytometry data were processed using FlowJo™ Software[2]. The cell population was first gated through forward and side scattering. The multiplets of cells are removed from the analyses. The means of FL1-A are used to determine the relative intracellular PCC-1.

Toxicity Assay

To evaluate the toxicity of the PCC-1 on ChoK1 cells, the viability of the cells is measured one day after different PCC-1 treatments. The cells are treated with PCC-1 at different concentrations (0, 0.3, 0.6, 1.2 μM) and for different periods (1, 2, 3, 5h). After PCC-1 treatments, the cells are washed with PBS for three times and incubated in growth media. After 24 h, the cells are washed and harvested by trypsinization. To include the detached

dead cells in the analyses, the growth media and PBS washes are combined with the cell solutions. The cells are pelleted with centrifugation (500 g, 5min). The cell pellets (about 50,000 cells) are then resuspended in 500 μL staining solution for 10 min at 37 °C and analyzed by a flow cytometer. The staining solution contains 2.5 μM SYTOX Green (green cell-impermeable nucleic acid stain) and 5 μM SYTO 59 (red cell-permeable nucleic acid stain) in L-15. SYTOX Green-positive cells are considered to be dead as the stain can only enter membrane-compromised cells. SYTO 59 is employed to reliably identify cells regardless of shape abnormality. The cell viability is calculated by taking the percentage of SYTOX Green -negative population in the SYTO 59 positive population.

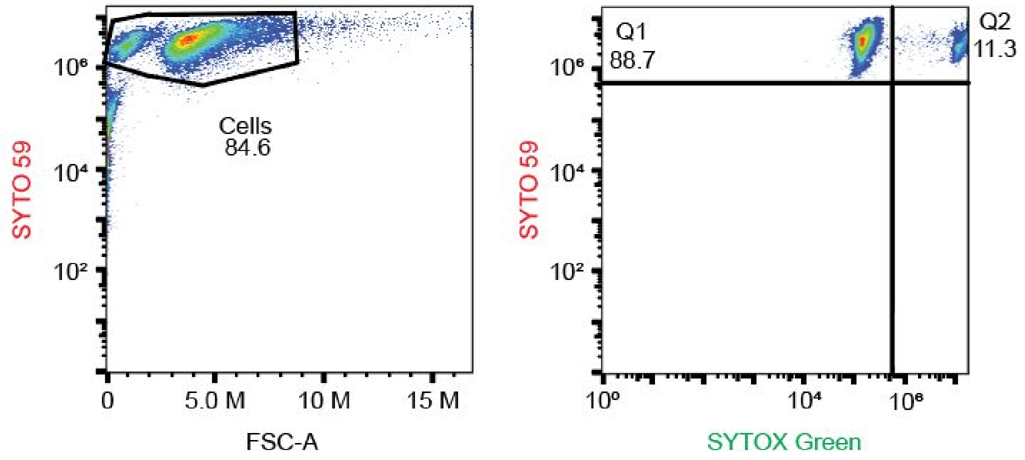

Figure S9. Processing of flow cytometry data to obtain cell viability based on the SYTO 59 and SYTOX Green staining in FlowJo™ Software.

Cell Proliferation Assay

The proliferation progress of the cells after PCC-1 treatment is monitored utilizing CellTrace™ CFSE Cell Proliferation Kit (CFSE: 5,6-carboxyfluorescein diacetate succinimidyl ester). One day before the experiment, 0.5 M cells /well CHO-K1 cells are first plated into a 6-well plate. After an overnight growth, the cells are firstly treated with CFSE (10 μM) in PBS (1 mL) for 10 min. The CFSE-labelled cells are treated with PCC-1 of different concentrations for 2 h. After the treatment, the cells are washed and

harvested from the plate by trypsinization. The cell concentration of the obtained cell suspension and the intracellular fluorescence (Day 0) are determined by a flow cytometer. Then, based on the cell concentration, the cells are replated into three different 48-well plates in the densities of 50,000 cells/well (Plate 1), 30,000 cells/well (Plate 2), and 10, 000 cells/well (Plate 3), respectively, for Day 1, Day 2 and Day 3 analyses. One day after the treatment, the cells in Plate 1 are harvested by trypsinization and analyzed by a flow cytometer for intracellular fluorescence (Day 1). On Day 2, the cells in Plate 2 are harvested and analyzed by a flow cytometer for cell concentrations and intracellular fluorescence (Day 2). Based on the cell concentration of each well, the cells are further replated into Plate 4, with 30,000 cells/well for Day 4 analysis. On Day 3, the cells in Plate 3 are harvested and analyzed by a flow cytometer for cell concentrations and intracellular fluorescence (Day 3). Then Plate 5 are prepared by seeding 30,000 cells/well into a new 48-well plate for day 5 analyses. On Day 4, the cells in Plate 4 are harvested by trypsinization and analyzed by a flow cytometer for intracellular fluorescence (Day 4). On Day 5 after the treatment, cells in Plate 5 are harvested and analyzed by a flow cytometer (Day 5).

The obtained flow cytometry data (Day 0 - 5) are analyzed and fit using cell proliferation model in the FlowJo™ Software to obtain proliferation parameters. The cell population is first gated through forward and side scattering. The multiplet of cells are removed from the analyses though FSC-A vs FSC-H plot gating. In the cell proliferation model, undivided population is defined as the cell population with CFSE fluorescence without any division. To model the division of cells from Day 0 to Day 1, the fluorescence intensities of cells at Day 0 is referred as the undivided cells for the Day 1 proliferation model. Similarly, to model the division of cells from Day 1 to Day 3, the fluorescence intensities of cells at Day 1 is referred as undivided cells for the Day 3 proliferation modelling. The same data process strategy is applied to Day 4 data (Day 2 as reference) and Day 5 data (Day 3 as reference) for optimal fitting into the model. From the modelling results, the division indexes are used to characterize the influence of PCC-1 on cell proliferation.

Cell Cycle analysis

Cell cycle analyses are conducted on PCC-1-treated CHO-K1 cells. These analyzes are conducted on the second day and the fourth day after PCC-1 treatments. One day before the experiment, CHO-K1 cells are first plated into a 6-well plate (0.5 M cells /well). In the 6-well plate, the CHO-K1 cells are treated with PCC-1 (0.3, 0.6 and 1.2 μM) for 2 h before they are washed and harvested by trypsinization. The harvested cells are counted and replated into two 12-well plates with the densities of 250,000 cells/well (Plate 5) and 100,000 cells /well (Plate 6). Cell cycle analyses are conducted on cells in Plate 5 on day 2 and in Plate 6 on day 4 after the PCC-1 treatment. The cell cycle analyses protocol utilized in this work is modified from reported protocols[3-5] that used SYTOX Green as the DNA stain and ethanol as the cell permeabilizing reagent. The staining solution is freshly made and contains 5 μM of SYTOX Green and 0.1 mg/mL RNAse A in PBS.

In a typical experiment, the cells harvested from the 12-well plates are first washed by warmed PBS (1 mL) and then by ice-cold PBS (1 mL) before they are permeabilized by ethanol. After washing, ice-cold PBS (0.3 mL) is added to the cell pellet and the cells are resuspended by pipetting. To the cell suspension, ice-cold absolute ethanol (0.7 mL) was added dropwise and quickly mixed before transferring to an ice bath. After a 2 h incubation, the permeabilized cells were pelleted (800 g, 5 min) and resuspended in PBS (0.5 mL), after which the cells were pelleted to remove leftover ethanol. To stain the permeabilized cells, a warmed staining solution (0.5 mL) was used to resuspend the cell pellet. The staining was at 37°C for at least 30 min. After the staining, the cells were analyzed by a flow cytometer. The acquired data were analyzed and modelled by FlowJo™ Software.

Absorbance and Fluorescence Analysis of H3PTH, PCC-1 and MB@PCC-1

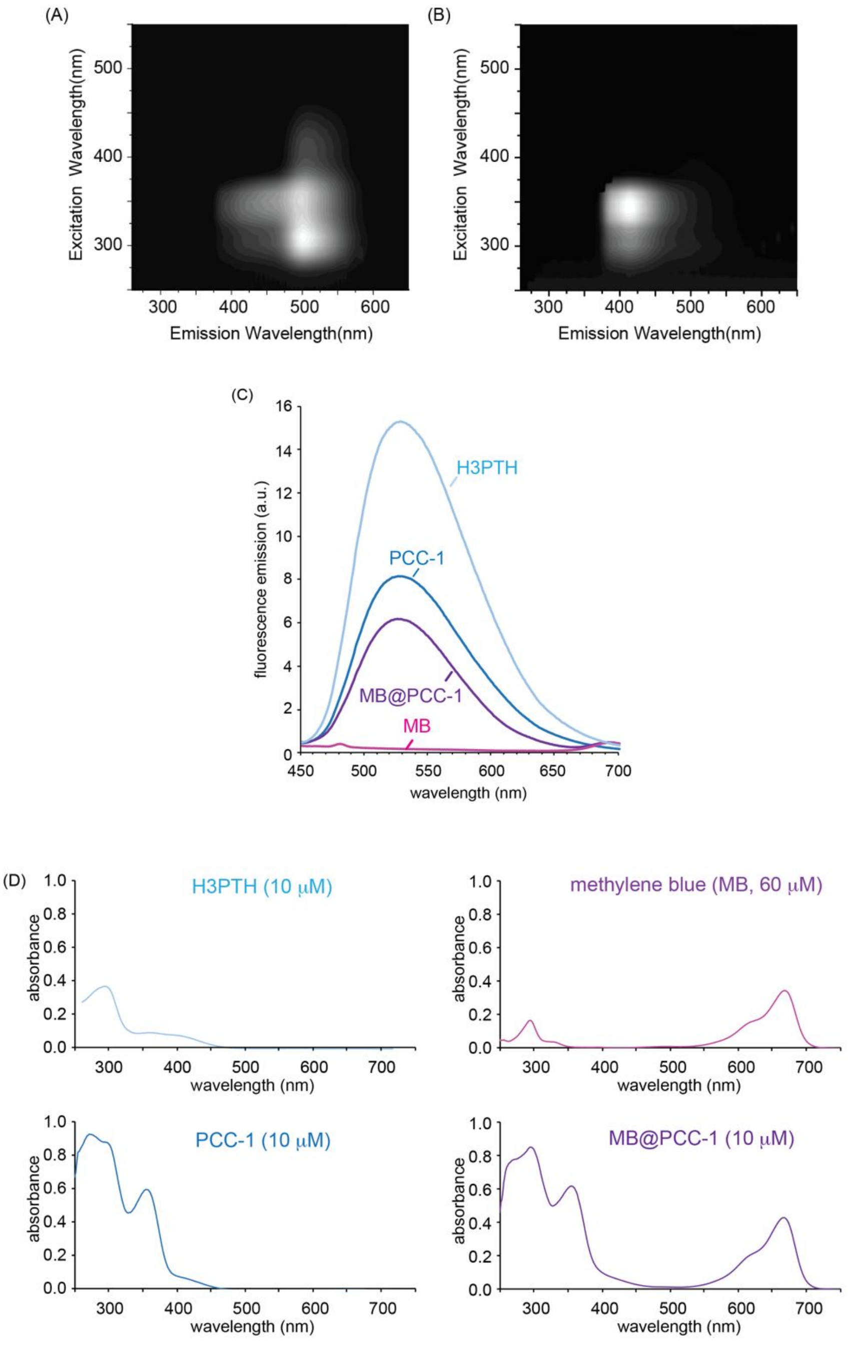

Figure S10. Fluorescence emission and absorbance of H3PTH, PCC-1, MB@PCC-1. A) Excitation-Emission matrix of PCC-1 (1.2 μM) in PBS. A) Excitation-Emission matrix of H_3PTH (10 μM) in PBS. C) Fluorescence Emission of H3PTH (80 μM), PCC-1 (10 μM), MB@PCC-1 (10 μM), and MB (60 μM). The fluorescence spectra were collected on Horiba Fluorescence Spectrophotometer. Excitation slit 5 nm, emission slit 5 nm. Excitation wavelength was 420 nm (corresponding to the excitation wavelength of the CPF filter - 420-450nm -

used for microscopy). D) Absorbance spectra of H3PTH, methylene blue, PCC-1, and MB@PCC-1 at the concentrations indicated in DMSO.

Results and Interpretation: The fluorescence properties of H3PTH have been reported.[6] The extinction coefficient of H3PTH in DMSO is 1.53×10^{3} $mL \cdot mol^{-1} \cdot cm^{-1}$ at 460 nm. The quantum yield of H3PTH was determined to be 0.18. Based on the results presented in Figure S10, the fluorescence emission of the ligand decreases by close to 50% in the context of PCC-1 (accounting for 8 H3PTH present per nanocage). Addition of MB and the formation of MB@PCC-1 causes an additional partial quenching.

NMR spectroscopy analysis of MB encapsulation by PCC-1

Proton Nuclear magnetic resonance (NMR) spectroscopy was performed on PCC-1, MB, and MB@PCC-1. Spectra were collected on AVANCE NEO 400 using DMSO-d6 as a solvent (solvent peak: 2.50 ppm, water peak: 3.30 ppm). For each spectrum, 32 scans were acquired, with a relaxation delay of 1s, and acquisition time of 3.2 sec, a transmitter frequency of 6.175 ppm, a Spectral width of 19.9947 ppm. Spectra were recorded at 6 mM MB, 1 mM PCC-1, 1 mM MB@PCC-1. NaOD (10 mM) was used to induce decomposition of PCC-1. A reference spectrum for MB with peak assignments is available at https://www.chemicalbook.com/SpectrumEN_61-73-4_1HNMR.htm.

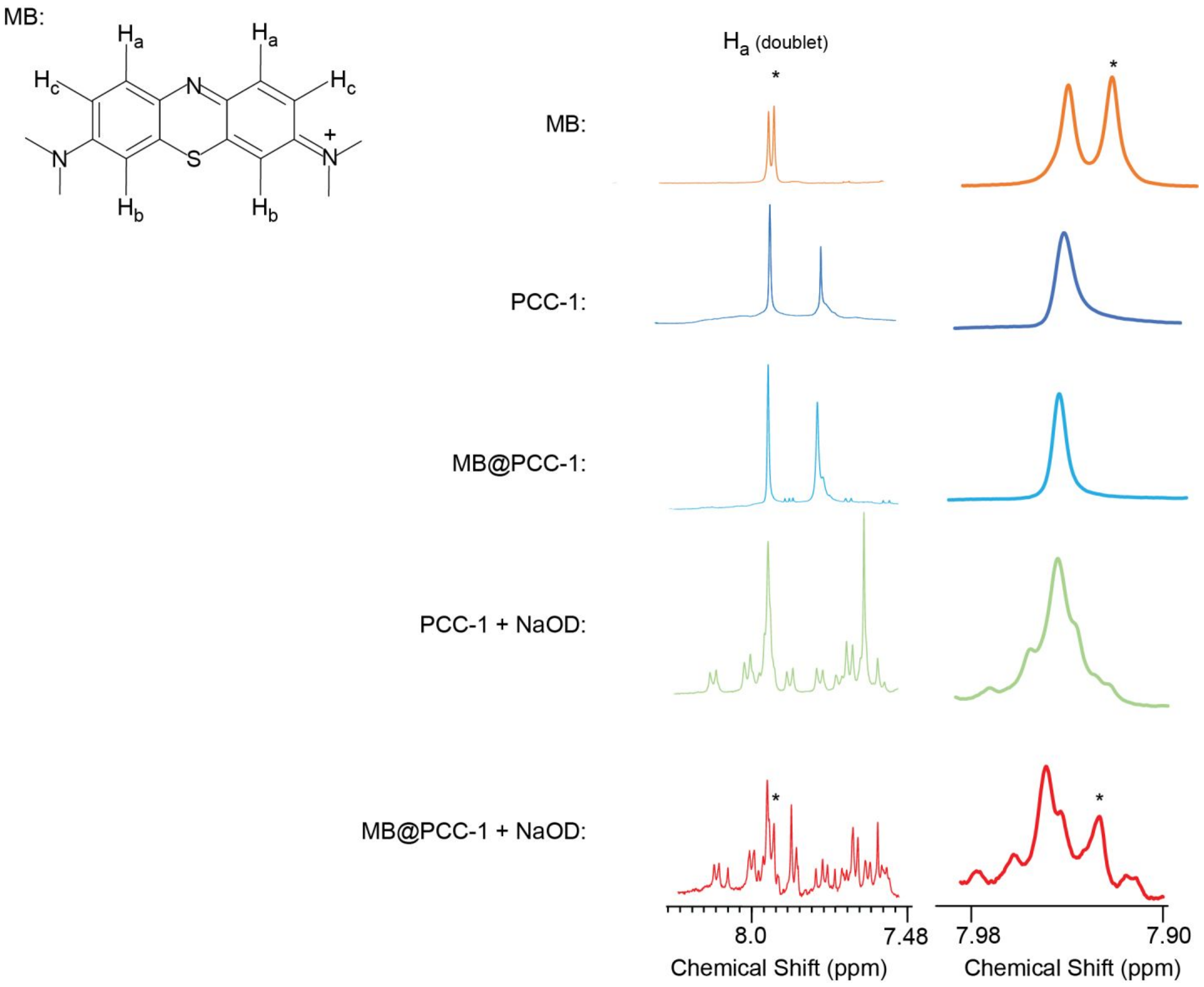

Figure S11. NMR spectra of MB, PCC-1, MB@PCC-1 before and after decomposition with NaOD. The chemical shift region for the aromatic Ha protons is displayed.

Results and Interpretation: PCC-1 and MB (Ha doublet 7.8 ppm, Hb singlet 7.4 ppm, Hc doublet 7.4 ppm, CH3 singlet 3.35 ppm) have overlapping resonances. The upfield component of the Ha doublet of MB is resolved from confounding resonances of PCC-1 and can serve as an unequivocal reporter of MB dynamics and environment. Results are presented in Figure S11, with the Ha resonance labeled with an asterix. In MB@PCC-1, this resonance is absent. Please note that one may expect that proton resonance of the MB guest within the PCC-1 host would move upfield due to aromatic shielding effect, as

observed in a previous study.[7] However, we do not detect a shift for any of the MB peaks. Specifically, the MB@PCC-1 spectrum is identical to that of PCC-1 in the range 9.0-0.0 ppm, with the MB resonances being instead absent (interpretation is confounded by overlap with PCC-1 and with solvent). Since the PCC-1 cage itself gives relatively narrow resonance line widths, consistent with the anticipated tumbling time in the low ns regime, we propose that the absence of the MB resonance is likely due to exchange broadening effects. It is well established by theory and experiment that nanometer scale confinement significantly slows rotational and translational dynamics of small molecules, often by many orders of magnitude.[8] Grossly slowed interconversion between the various states of multiple MB molecules within each PCC-1 cage can then lead to incomplete averaging on the chemical shift time scale and broadening of resonances arising from the various environments, orientations and dynamics of MB within the cage into the baseline. It is worth noting the decomposition of PCC-1 with NaOD restore the MB Ha resonance, indicating that MB is present in the MB@PCC-1 sample and that disassembly of PCC-1 releases free MB. If MB was bound to the surface of PCC-1, it would be expected to experience similar dynamics as the cage itself. Given that the resonances of PCC-1 are visible in the NMR spectrum, had MB been on the surface, its peaks would likely also be visible. The absence of MB peaks suggests a different dynamic environment, consistent with it being encapsulated within PCC-1 rather than being on the outside.

References

1. Fang, Y.; Lian, X.; Huang, Y.; Fu, G.; Xiao, Z.; Wang, Q.; Nan, B.; Pellois, J. P.; Zhou, H. C., Investigating Subcellular Compartment Targeting Effect of Porous Coordination Cages for Enhancing Cancer Nanotherapy. *Small* **2018,** *14* (47), e1802709.
2. *FlowJo™ Software for Windows Version 10.80*, Becton, Dickinson and Company: Ashland, OR, 2023.
3. Haase, S. B.; Reed, S. I., Improved Flow Cytometric Analysis of the Budding Yeast Cell Cycle. *Cell Cycle* **2002,** *1* (2), 117-121.
4. Haase, S. B., Cell Cycle Analysis of Budding Yeast Using SYTOX Green. *Current Protocols in Cytometry* **2003,** *26* (1), 7.23.1-7.23.4.
5. Delobel, P.; Tesnière, C., A Simple FCM Method to Avoid Misinterpretation in Saccharomyces cerevisiae Cell Cycle Assessment between G0 and Sub-G1. *PLOS ONE* **2014,** *9* (1), e84645.
6. Lin, H. Y.; Xiao, Z. F.; Le, K. N.; Yan, T. H.; Cai, P. Y.; Yang, Y. H.; Day, G. S.; Drake, H. F.; Xie, H. M.; Bose, R.; Ryan, C. A.; Hendon, C. H.; Zhou, H. C., Assembling Phenothiazine

into a Porous Coordination Cage to Improve Its Photocatalytic Efficiency for Organic Transformations. *Angew Chem Int Edit* **2022,** *61* (49).
7. Murase, T.; Nishijima, Y.; Fujita, M., Cage-catalyzed Knoevenagel condensation under neutral conditions in water. *J Am Chem Soc* **2012,** *134* (1), 162-4.
8. Alba-Simionesco, C.; Coasne, B.; Dosseh, G.; Dudziak, G.; Gubbins, K. E.; Radhakrishnan, R.; Sliwinska-Bartkowiak, M., Effects of confinement on freezing and melting. *J Phys Condens Matter* **2006,** *18* (6), R15-68.
